# Comparison of the connectivity of the posterior intralaminar thalamic nucleus and peripeduncular nucleus in rats and mice

**DOI:** 10.3389/fncir.2024.1384621

**Published:** 2024-04-26

**Authors:** Hui-Ru Cai, Sheng-Qiang Chen, Xiao-Jun Xiang, Xue-Qin Zhang, Run-Zhe Ma, Ge Zhu, Song-Lin Ding

**Affiliations:** ^1^Key Laboratory of Neuroscience, School of Basic Medical Sciences, Guangzhou Medical University, Guangzhou, China; ^2^Department of Psychology, School of Health Management, Guangzhou Medical University, Guangzhou, China; ^3^Allen Institute for Brain Science, Seattle, WA, United States

**Keywords:** connections, amygdala, posterior striatum, hypothalamus, auditory thalamus, ectorhinal cortex, inferior colliculus, parabigeminal nucleus

## Abstract

The posterior intralaminar thalamic nucleus (PIL) and peripeduncular nucleus (PP) are two adjoining structures located medioventral to the medial geniculate nucleus. The PIL-PP region plays important roles in auditory fear conditioning and in social, maternal and sexual behaviors. Previous studies often lumped the PIL and PP into single entity, and therefore it is not known if they have common and/or different brain-wide connections. In this study, we investigate brain-wide efferent and afferent projections of the PIL and PP using reliable anterograde and retrograde tracing methods. Both PIL and PP project strongly to lateral, medial and anterior basomedial amygdaloid nuclei, posteroventral striatum (putamen and external globus pallidus), amygdalostriatal transition area, zona incerta, superior and inferior colliculi, and the ectorhinal cortex. However, the PP rather than the PIL send stronger projections to the hypothalamic regions such as preoptic area/nucleus, anterior hypothalamic nucleus, and ventromedial nucleus of hypothalamus. As for the afferent projections, both PIL and PP receive multimodal information from auditory (inferior colliculus, superior olivary nucleus, nucleus of lateral lemniscus, and association auditory cortex), visual (superior colliculus and ectorhinal cortex), somatosensory (gracile and cuneate nuclei), motor (external globus pallidus), and limbic (central amygdaloid nucleus, hypothalamus, and insular cortex) structures. However, the PP rather than PIL receives strong projections from the visual related structures parabigeminal nucleus and ventral lateral geniculate nucleus. Additional results from Cre-dependent viral tracing in mice have also confirmed the main results in rats. Together, the findings in this study would provide new insights into the neural circuits and functional correlation of the PIL and PP.

## 1 Introduction

The posterior intralaminar nucleus (PIL) and peripeduncular nucleus (PP) of the posterior thalamus are located ventromedial to the medial geniculate nuclear complex (MG), which usually includes the dorsal, ventral, and medial subdivisions (MGd, MGv, and MGm, respectively). Compared to the MG, the PIL is a triangular structure in coronal sections while the PP is a band-like structure located ventrolateral to the PIL. The boundaries between the PIL and PP are difficult to place in Nissl-stained sections and in literature, there is disagreement about the exact boundaries and extent of the PIL and PP in rats (Linke, 1999a; Linke et al., 2000; Lanuza et al., 2008; Márquez-Legorreta et al., 2016). Some authors treat the PIL/PP together as a single entity (i.e., PIL or PP) (Factor et al., 1993; Keller et al., 2022; Valtcheva et al., 2023; Leithead et al., 2024). Furthermore, some authors further include the MGm and/or suprageniculate nucleus (SG) into this nuclear complex (Campeau and Watson, 2000, Cservenák et al., 2013). In connectional studies, tracer injections are often involved in all or parts of this complex while the resulting connections are often claimed to belong to the PIL or PP (e.g., Arnault and Roger, 1987; Campeau and Watson, 2000). This PIL-PP region receives projections from both the superior colliculus (SC) and inferior colliculus (IC) (Linke, 1999a), and projects to the lateral nucleus of amygdala (La), striatum, and auditory cortex (Smith et al., 2019). This region also receives auditory, visual, somatosensory, vestibular, and nociceptive inputs from the midbrain and several brainstem regions (Bordi and LeDoux, 1994a,b; Benedek et al., 1997; Shi and Davis, 1999; Shiroyama et al., 1999; Linke, 1999a) and originates auditory thalamo-amygdalar and thalamo-hypothalamic (particularly the paraventricular nucleus, PaH) projections in rats (Herkenham, 1986; LeDoux et al., 1990; Campeau and Watson, 2000). The former projections mediate auditory fear conditioning (Romanski and LeDoux, 1992; Campeau and Davis, 1995; Boatman and Kim, 2006), while the latter appears to mediate the response to audiogenic stress (Campeau and Watson, 2000). In fact, many studies have shown that the thalamo-hypothalamic pathways are critical to social, sexual, and maternal behaviors (López and Carrer, 1982, 1985; Hansen and Köhler, 1984; Factor et al., 1993; Dobolyi et al., 2018; Valtcheva et al., 2023; Leithead et al., 2024). Interestingly, the region corresponding to human PP appears to be an effective target for deep brain stimulation in severe Parkinson’s disease (Stefani et al., 2007) although the authors appear misnamed the region, as pointed out by other authors (Zrinzo et al., 2007).

Given these important and different roles of the PIL-PP region, it is important to pinpoint the individual nuclei in this complex region to explore their common and distinct neural circuits and possible functional correlation. As a first step toward these goals, the present study mainly focuses on brain-wide efferent and afferent connections of the PIL and PP in rats. Our results have not only confirmed many connections of the PIL-PP region reported in previous studies but also revealed some novel connections and differences between the PIL and PP. These findings are also confirmed in mice with Cre-dependent anterograde tracing methods. Together, these results would provide an important anatomical basis for detailed functional and brain stimulation studies of the PIL and PP.

## 2 Materials and methods

### 2.1 Animals

Forty adult Sprague–Dawley rats of both sexes weighing 280–310 g (Beijing Vital River Laboratory Animal Technology Co., Ltd., Beijing, China) were used in this study. All the animals were placed in the same environment with suitable temperatures and controlled light, as well as free access to food and water. All surgery operations were performed under deep anesthesia to alleviate their pain. All experimental procedures were followed in accordance with the protocols that have been approved by the Institutional Animal Care and Use Committee of Guangzhou Medical University.

### 2.2 Surgery procedure and tracer injections

As previously described (Lu et al., 2020; Chen et al., 2021), the rats were anesthetized with intraperitoneal injection of 0.3% pentobarbital sodium (40 mg/kg). When the rear foot reflex and muscle tension of the rats disappeared, the rats were fixed on the stereotaxic apparatus, and the eyes of the rats were covered with paper towels to prevent the eyesight from being affected by the long-term irradiation of the operating lamp. After disinfection, the skin over the skull midline was cut to expose the bregma, lambda and the skull surface over the injection sites. The stereotaxic coordinates of the target brain regions were determined before the surgery according to the rat brain atlas (Paxinos and Watson, 2013). A small hole in the skull was drilled over the target region, and then 0.05–0.07 μL 10% biotinylated dextran amine (BDA) or 4% Fluoro-Gold (FG) was injected into the target region using a 1 μL Hamilton microsyringe connected to the microinjection pump to deliver the neuronal tracers. After the injection was completed, the needle was left in place for 10 min and then pulled out slowly to avoid leakage of the tracers to other areas. Finally, the incision was sutured with absorbable sutures and the skin was disinfected. The rat was then placed on the warming bed and returned to its cage after it woke up and could move freely.

### 2.3 Brain preparation

7–10 days after the completion of brain stereotaxic surgery, the rats were deeply anesthetized with an intraperitoneal injection of 0.3% pentobarbital sodium (80 mg/kg). Then, perfusion of 150 mL of 0.9% normal saline, 300 mL of 4% paraformaldehyde in 0.1 M phosphate buffered saline (PBS) were carried out in sequence as described previously (Lu et al., 2020; Chen et al., 2021). The rat brains were then removed from the cranial cavity and soaked in 4% paraformaldehyde solution overnight. Finally, the brains were cryoprotected in 15% and 30% sucrose solution in sequence for 3–4 days, until the brains sank to the bottom of the container. For cryo-sectioning, each brain was cut along the midline and divided into two hemispheres, after embedding in OCT. The brain tissue was cut coronally with a cryostat with a thickness of 40 μm, and all sections were collected in the order from the anterior to posterior levels. All the sections were stored in a cryoprotectant solution in a refrigerator at 4°C until use.

### 2.4 Nissl’s staining

The sections for Nissl-staining were first washed three times in PBS and then mounted on the adhesive glass slides and dried for 24 h. Next, the sections were placed in xylene and graded ethanol solutions (in the order of 100, 95, 85, and 70%) for 5 min each, then stained in 0.1% Cresyl Violet solution for 20 min and immersed in distilled water for 5 min. The sections were then dehydrated in 85, 95, and 100% ethanol for 5 min, and placed in xylene (twice, 5 min each). Finally, the sections were sealed with neutral gum and dried.

### 2.5 BDA tracing

The sections from the brains injected with BDA were stained according to a previously published method (Chen et al., 2021; Xiang et al., 2023). Briefly, the sections were first washed in 0.05 M PBS (at least three times, 5 min each), and incubated in the Triton solution (0.3% Triton X-100:0.05 M PBS = 3:1,000) at room temperature for 1 h. The sections were then incubated in a Streptavidin-Biotin complex solution (SABC kit, Boster Biological Technology) for 3 h at room temperature. After rinsing in 0.05 M PBS three times, the sections were visualized with 0.05 M PBS containing 0.05% 3, 3-diaminobenzidine (DAB) and 0.01% hydrogen peroxide. Finally, the sections were mounted on chrome alum and gelatin coated glass slides, dehydrated in gradient ethanol and xylene, and finally coverslipped.

### 2.6 FG tracing

Selected sections along the anterior–posterior levels of the hemisphere from the brains injected with FG were first rinsed and mounted on glass slides and then examined under an upright fluorescence microscope (Leica DM6B or Axio Observer7). Some other sections were stained with immunohistochemistry (IHC) for FG. For the IHC, the sections were washed in 0.05 M PBS (at least three times, 5 min each), and then incubated in 3% hydrogen peroxide for 10 min at room temperature (to remove endogenous peroxidase). After thorough rinses, the sections were blocked in 5% bovine serum albumin for 60 min at room temperature and then incubated in 0.05 M PBS containing 0.3% Triton X-100 and the primary antibody (anti-FG, AB153-I, 1:10,000, Sigma-Aldrich) overnight at 4°C. The next day, the sections were rewarmed at room temperature for 60 min, and then washed with 0.05 M PBS for four times, 5 min each. The sections were then soaked in the secondary antibody solution (Biotinylated goat anti-mouse/rabbit IgG, Boster Biotech) and incubated for 60 min at room temperature. Following three times rinses with 0.05 M PBS, the sections were incubated in the streptavidin-biotin complex solution (SABC kit, Boster Biotech) at room temperature for 60 min. After rinsing, the sections were then soaked in 0.05 M PBS containing 0.05% DAB and 0.01% hydrogen peroxide and incubated at room temperature until suitable color of staining appears. Finally, 0.01 M PBS was used to stop the color development. After mounting onto the grass slides and drying, the sections were placed in gradient ethanol and xylene, and finally sealed with neutral resin.

### 2.7 Cell counts and statistics of FG-labeled neurons in rats

To quantify and compare the numbers of FG-labeled neurons following FG injections in the PIL or PP, sequential coronal sections throughout the brains (six cases, three for PIL, three for PP injections) were selected, and FG labeled cells in different brain regions were counted for each of six brains. Similarly, after FG injection in the ventromedial nucleus of hypothalamus (VMH), FG-labeled neurons in the PIL and PP were also counted to compare the numbers of cells in the PIL and PP that projects to the VMH (three cases). Cell counts was performed using Image J, and Multiple T-tests were used to compare the numbers of labeled cells resulted from the PIL and PP injections. For comparison of the cell counts in the PIL and PP after VMH injections, two independent samples paired T-tests were employed.

### 2.8 Analysis of the mouse gene expression data from Allen Institute

To identify some molecular markers of the PIL and PP in mice, we searched the Allen Mouse Atlas^1^ and found some genes with their expression mainly in the PP or PIL + PP but not or faint in adjoining regions. We matched and compared the expression intensity of four genes in sections that are closely adjacent to Nissl-stained sections to further confirm the locations and extent of the PIL and PP.

### 2.9 Analysis of the mouse connectivity data from Allen Institute

To explore if main connections of the mouse PIL and PP are similar to those in the rats, we searched the Allen Mouse Connectivity Atlas^2^ and found four cases with anterograde viral tracer injections restricted in the PIL (two Cre-line cases) or in both PIL and PP (one Cre-line case and one wild-type case). We then analyzed and compared the main target regions of the PIL and PIL + PP efferent projections. Additionally, we also found and examined several cases with the viral tracers restricted in the VMH, LDTg, and Ce and then compared the overall density of the labeled axon terminals in the PIL and PP resulted from the injections in these three structures.

### 2.10 Image acquisition and processing

The images from the sections containing FG labeling were obtained using an epifluorescent microscope (Leica DM6B or Axio Observer7). The images from BDA-stained sections were acquired with a section scanner (Aperio CS2, Leica). The images from Allen datasets^3^ were downloaded from the website. All the images shown in the Result section were processed with Adobe photoshop for image cropping, brightness and contrast adjustment, image composing, and structural annotation.

## 3 Results

### 3.1 General location and cytoarchitecture of the PIL and PP

**Figure 1:**
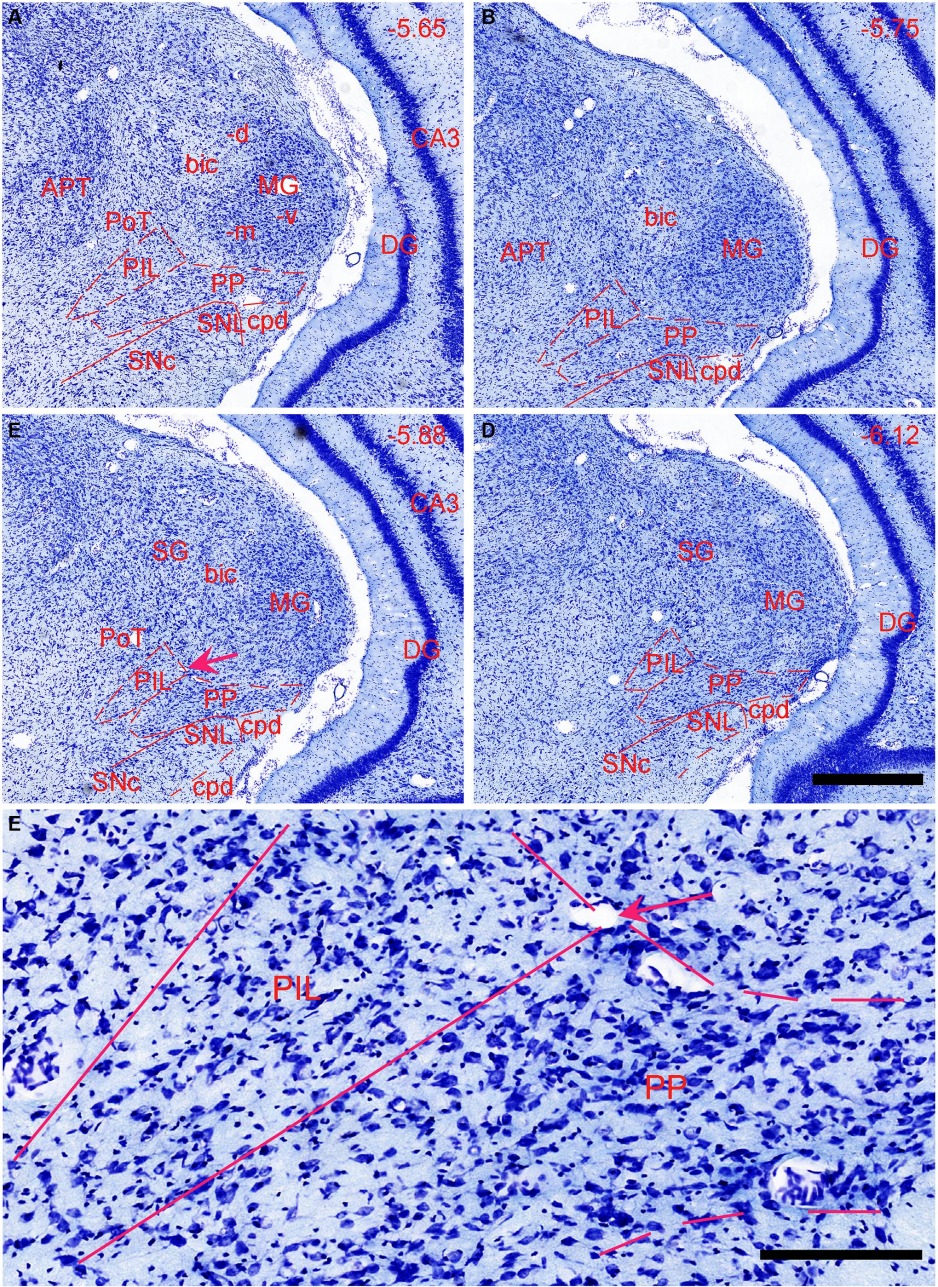
Location and cytoarchitecture of the rat posterior intralaminar nucleus (PIL) and peripeduncular nucleus (PP) revealed with Nissl staining. **(A–D)** PIL and PP in sequential anterior **(A)** to posterior **(D)** coronal sections. Overall, the PIL is located dorsomedial to the PP while both are located ventromedial to the MGm. The PP immediately overlies the lateral substantia nigra (SNL) and cerebral peduncle (cpd). Approximate bregma coordinates are indicated at the top right corner of each panel. **(E)** A high magnification view of the PIL and PP from panel **(C)**, showing the cytoarchitecture and overall cell sizes of both structures. Note that the PIL appears to contain many smaller cells than the PP does. The arrows in panels **(C,E)** point to the same location. Bars: 500 μm in panel **(D)** for panels **(A–D)**; 125 μm in panel **(E)**.

In previous studies, the PIL, PP, and MGm, were often grouped as a single nucleus of the auditory thalamus (e.g., Deacon et al., 1983; Smith et al., 2006). The PIL is a triangular structure of the posterior thalamus in coronal sections and is located ventromedial to the MGm (Figures 1A–D). In Nissl-stained sections, the PIL contains many smaller neurons as compared to the PP, which has more larger neurons (Figure 1E). MGm is mostly composed of larger cells and is located ventral to the brachium of the inferior colliculus (bic; the cell-sparse region) and medial to the ventral MG (MGv) (Figure 1A). The borders between the PIL and PP or MGm are difficult to appreciate in Nissl preparations but overall, the PIL is located dorsal to the PP, which is a band-like structure immediately adjoining the underlying substantia nigra, lateral part (SNL), and cerebral peduncle (cpd) (see Figures 1A–D). The locations of the PIL and PP in four representative anterior–posterior sections are shown in Figures 1A–D, respectively.

**Figure 2:**
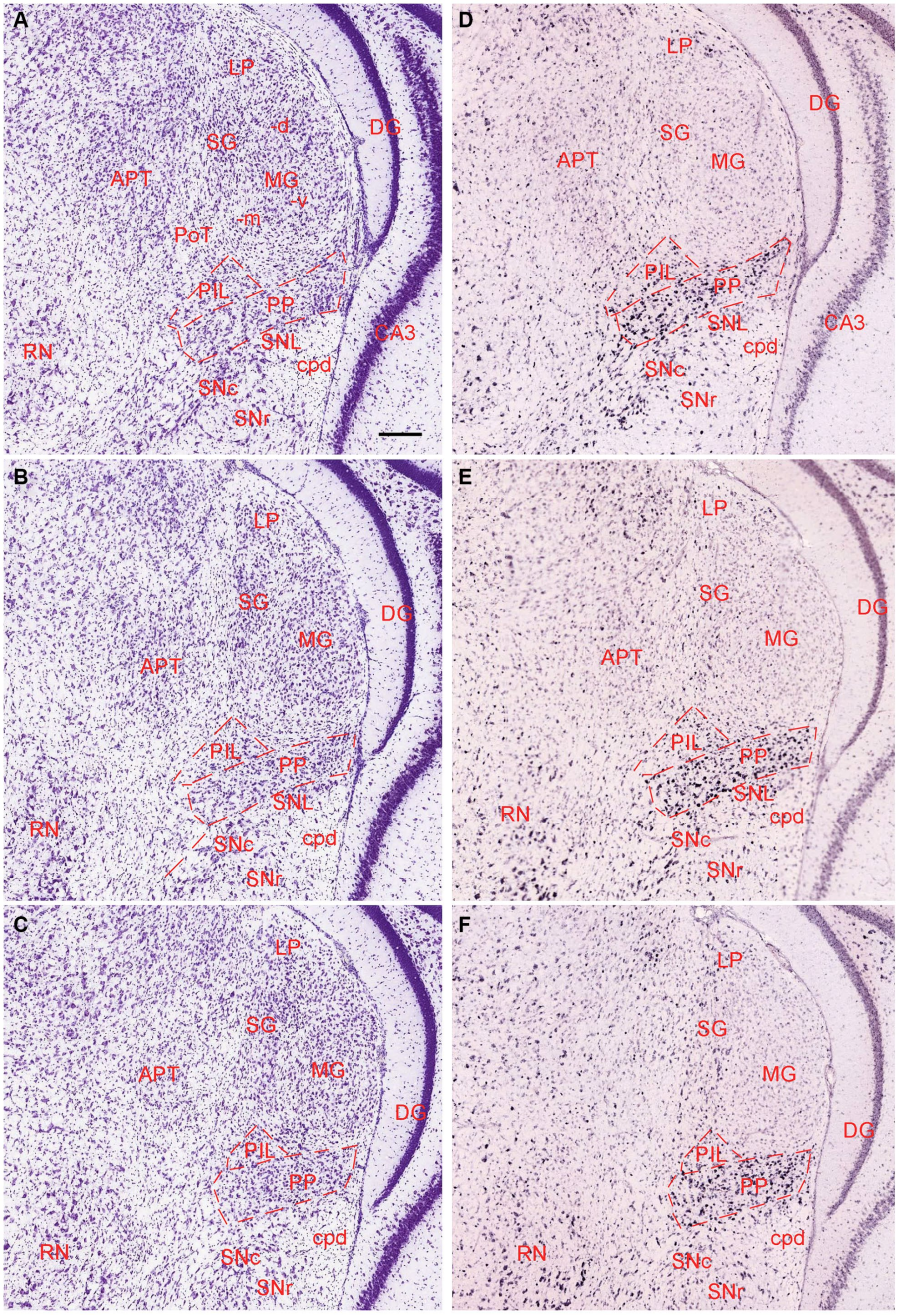
Location and architecture of the mouse PIL and PP. **(A–C)** Location and cytoarchitecture of the mouse PIL and PP in sequential anterior **(A)** to posterior **(C)** Nissl-stained sections. Note that the location of the PIL and PP in the mouse is very similar to that in rats. **(D–F)** Expression of the gene *Tppp3* in mouse PIL and PP in sequential anterior **(D)** to posterior **(F)** sections. Panels **(A,D,B,E)**, as well as panels **(C,F)** are closely adjacent coronal sections, respectively. *Tppp3* is predominantly expressed in the PP with much less expression in the PIL. Bar: 210 μm in panel **(A)** for all panels.

**Figure 3:**
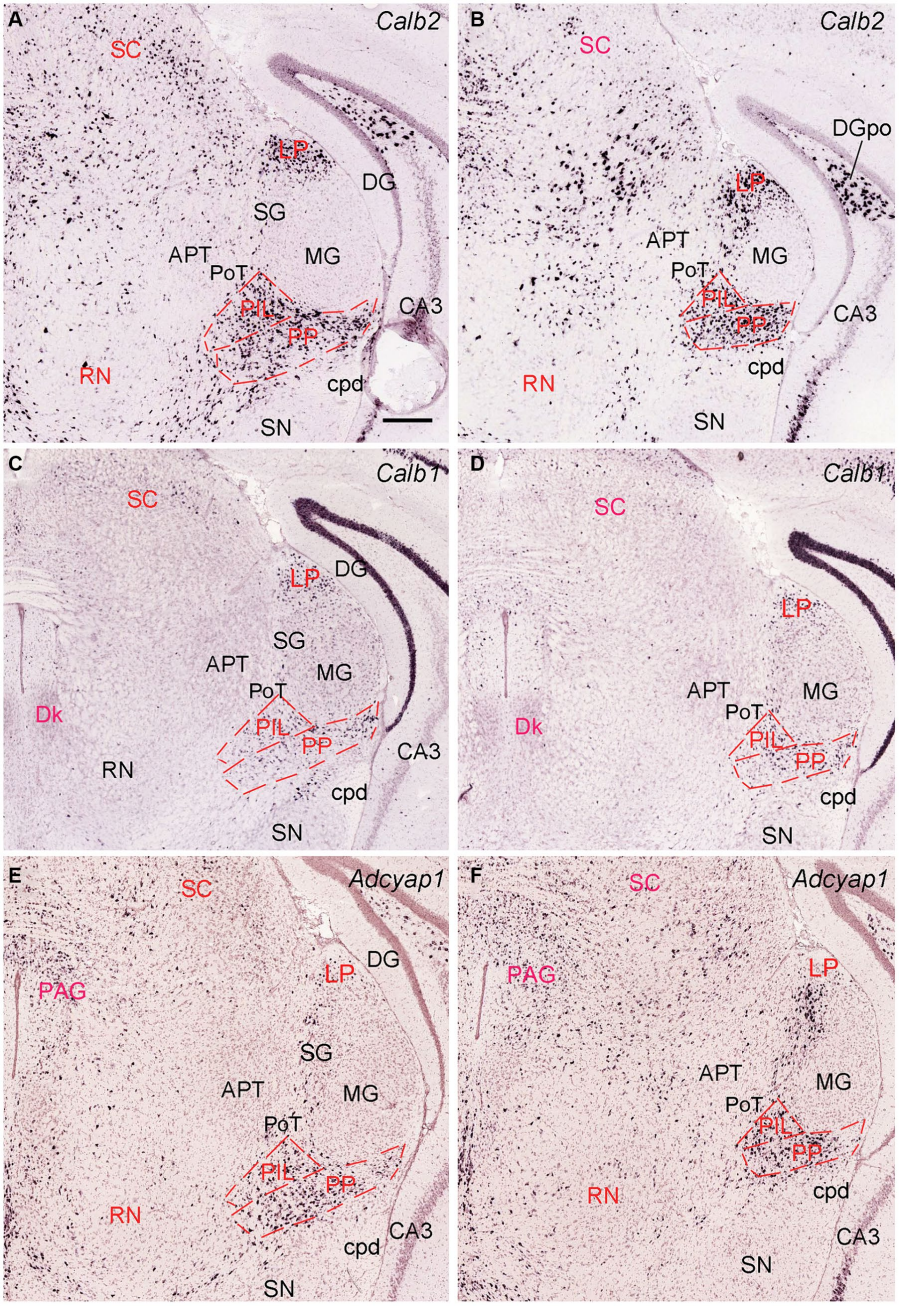
Molecular markers of the mouse PIL and PP. **(A,B)** Expression of the gene *Calb2* in the PIL and PP. **(C,D)** Expression of the gene *Calb1* in the PIL and PP. **(E,F)** Expression of the gene *Adcyap1* in the PIL and PP. Panels **(A–D)**, as well as panels **(E,F)** are representative anterior and posterior coronal sections, respectively. Note that these genes are strongly expressed in both PIL and PP with only a little expression in the adjoining PoT and MG. Strong expression of Calb2 and Calb1 is also seen in the LP (i.e., LP-Pul). Bar: 210 μm in panel **(A)** for all panels.

The general locations and cytoarchitectonic features of the PIL and PP in mice (Figures 2A–C) are similar to those in rats (Figures 1A–D). Additionally, by searching the mouse gene expression dataset from the Allen Institute, we find that the gene *tubulin polymerization-promoting protein family member 3 (Tppp3)* is strongly expressed in the PP (Figures 2D–F). This expression pattern makes the PP stand out from adjoining PIL, SNL, and MG, all of which display weak *Tppp3* expression (Figures 2D–F). The location and extent of the PP identified with *Tppp3* expression pattern is consistent with those identified in closely adjacent Nissl-stained sections (Figures 2A–C). Finally, we also find some genes that are expressed in both PIL and PP such as calbindin 2 (*Calb2*; Figures 3A,B), calbindin 1 (*Calb1*; Figures 3C,D), and *adenylate cyclase activating polypeptide 1* (*Adcyyap1*; Figures 3E,F).

### 3.2 Brain-wide efferent projections of the PIL and PP in rats

**Figure 4:**
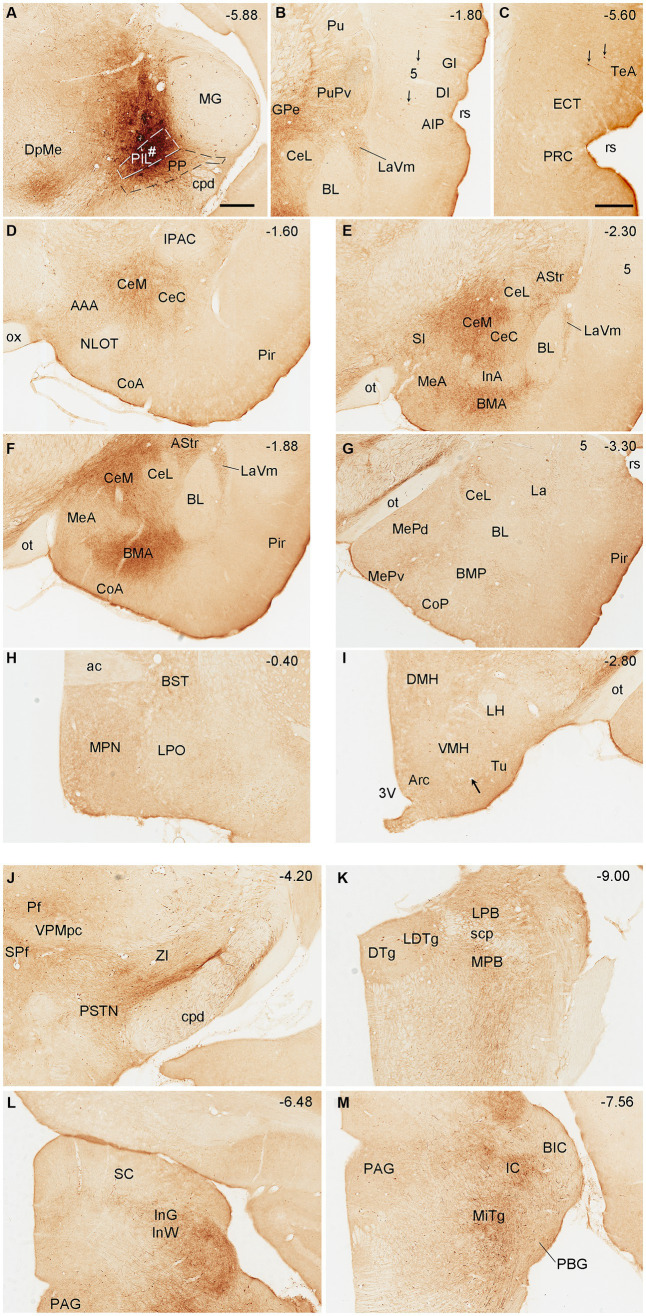
Efferent projections of the rat PIL revealed with BDA. **(A)** One biotinylated dextran amine (BDA) injection site (#) is mostly restricted to the PIL without involvement in the underlying PP. **(B–M)** BDA labeled axonal terminals in the insular cortices (AIP, DI, and GI), PuPv, GPe, and LaVm **(B)**, in the PRC, ECT, and TeA **(C)**, in the AAA, CeM, CeC, and CoA **(D)**, in the SI, CeM, CeC, BMA, MeA, AStr, and LaVm **(E,F)**, in the MeP, BMP, and La **(G)**, in the MPN and BST **(H)**, in the VMH and Tu **(I)**, in the Pf, SPf, PSTN, and ZI **(J)**, in the LDTg, LPB, and MPB **(K)**, in the SC and PAG **(L)**, and in the IC and MiTg **(M)**. Note that strong terminal labeling is mainly seen in PuPv, GPe, AStr, CeM, LaV, BMA, SC, IC, and MiTg. Only weak or faint terminal labeling is observed in other regions including MPN and VMH (see high magnification view in Figure 6A). In addition, a few labeled neurons are seen in layer 5 of the insular cortex **(B)** and TeA **(C)**, and in the MPB and LDTg **(K)**. Approximate bregma coordinates are indicated at the top right corner of each panel. Bar: 500 μm in panel **(A)** for all panels except panel **(C)**; 500µm in **(C)**.

To reveal brain-wide efferent projections of the PIL and PP in the rats, we inject the tracer BDA into the PIL (three cases) or PP (four cases), and in three additional cases, the injections are involved in both PIL and PP. Evaluation of the injection sites is based on the rat brain atlas (Paxinos and Watson, 2013) and our Nissl-stained sections (e.g., Figures 1A–D). When a BDA injection is restricted to the PIL (e.g., Figure 4A), labeled axon terminals are mostly observed in subcortical regions with no or faint labeling in the insular (AIP, DI, and GI; Figure 4B), perirhinal (PRC), and ectorhinal (ECT) cortices (Figure 4C). The subcortical regions that contain many labeled axon terminals include the posteroventral putamen (PuPv; Figure 4B) and adjoining external globus pallidus (GPe; Figure 4B), medial part of the ventral lateral amygdaloid nucleus (LaVm; Figures 4B,E,F), medial central amygdaloid nucleus (CeM; Figures 4D–F), anterior basomedial amygdaloid nucleus (BMA; Figures 4E,F), amygdalostriatal transition area (AStr; Figures 4E,F), ventral parafascicular nucleus (Pf), subparafascicular nucleus (SPf), parasubthalamic nucleus (PSTN) and zone incerta (ZI) (Figure 4J), superior colliculus (SC, the middle layers InG and InW; Figure 4L), inferior colliculus (IC), and microcellular tegmental nucleus (MiTg; Figure 4M). It is noted that only faint terminal labeling is seen in anterior and posterior medial amygdala nucleus (MeA and MeP; Figures 4F,G), medial preoptic nucleus of hypothalamus (MPN; Figure 4H), ventromedial nucleus of hypothalamus (VMH; Figure 4I), medial and lateral parabrachial nuclei (MPB and LPB; Figure 4K), and periaqueductal gray (PAG; Figure 4L). It is also obvious that no labeling is detected in the basolateral amygdaloid nucleus (BL; Figures 4E,F). In addition, some BDA-labeled cell bodies are seen in the LPB-MPB and the lateral dorsal tegmental nucleus (LDTg; Figure 4K) but not in the parabigeminal nucleus (PBG; Figure 4M).

**Figure 5:**
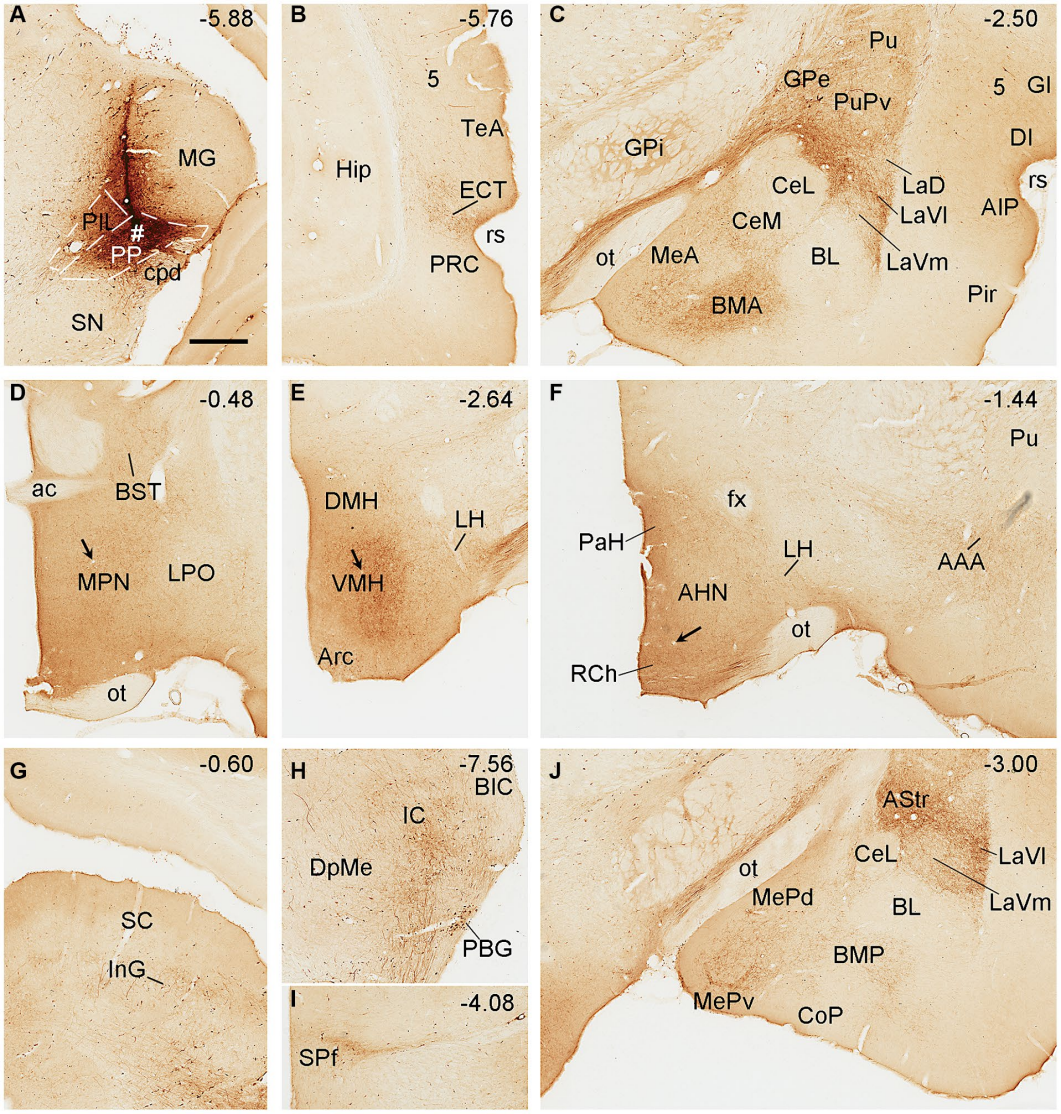
Efferent projections of the rat PP revealed with BDA. **(A)** One BDA injection site (#) is mainly restricted to the PP with slight involvement in the overlying PIL. **(B–J)** BDA labeled axonal terminals in the ECT **(B)**, PuPv, GPe, BMA, La, and Me **(C)**, MPN and BST **(D)**, VMH **(E)**, AHN, RCh, and AAA **(F)**, SC **(G)**, IC **(H)**, SPf **(I)**, and AStr, MeP, and LaVl **(J)**. Note that stronger terminal labeling is mainly seen in ECT, PuPv, GPe, AStr, LaV, Me, BMA, AAA, and IC, as well as in the MPN and VMH (see high magnification views in Figures 6B,C). Weaker terminal labeling is also detected in other hypothalamic regions such as the RCh and AHN (**F**; with high power view in Figure 6D). It is also noted that layer 5 of the insular cortex **(C)** as well as the PBG **(H)** contain BDA labeled cell bodies. Approximate bregma coordinates are indicated at the top right corner of each panel. Bar: 500 μm in panel **(A)** for all panels.

Biotinylated dextran amine injections in the PP (e.g., Figure 5A) result in labeled axon terminals in the ECT (Figure 5B), PuPv-GPe (Figure 5C), BMA (Figure 5C), AStr (Figures 5C,J), Me (Figures 5C,J), lateral part of the ventral lateral amygdaloid nucleus (LaVl; Figures 5C,J), MPN (Figure 5D), bed nucleus of stria terminalis (BST; Figure 5D), VMH (Figure 5E), anterior hypothalamic nucleus (AHN; Figure 5F), retrochiasmatic area (RCh; Figure 5F), anterior amygdaloid area (AAA; Figure 5F), SC (Figure 5G), IC (Figure 5H), SPf (Figure 5I), and BMP (Figure 5J). Therefore, compared to the PIL, the PP clearly projects to the ECT, MeP, MPN, VMH, and AHN, and these regions do not appear to receive strong inputs from the PIL. In addition, some BDA retrogradely labeled cells are found in layer 5 of the temporal (Figure 5B) and insular (Figure 5C) cortices while many labeled neurons are seen in the PBG (Figure 5H).

**Figure 6:**
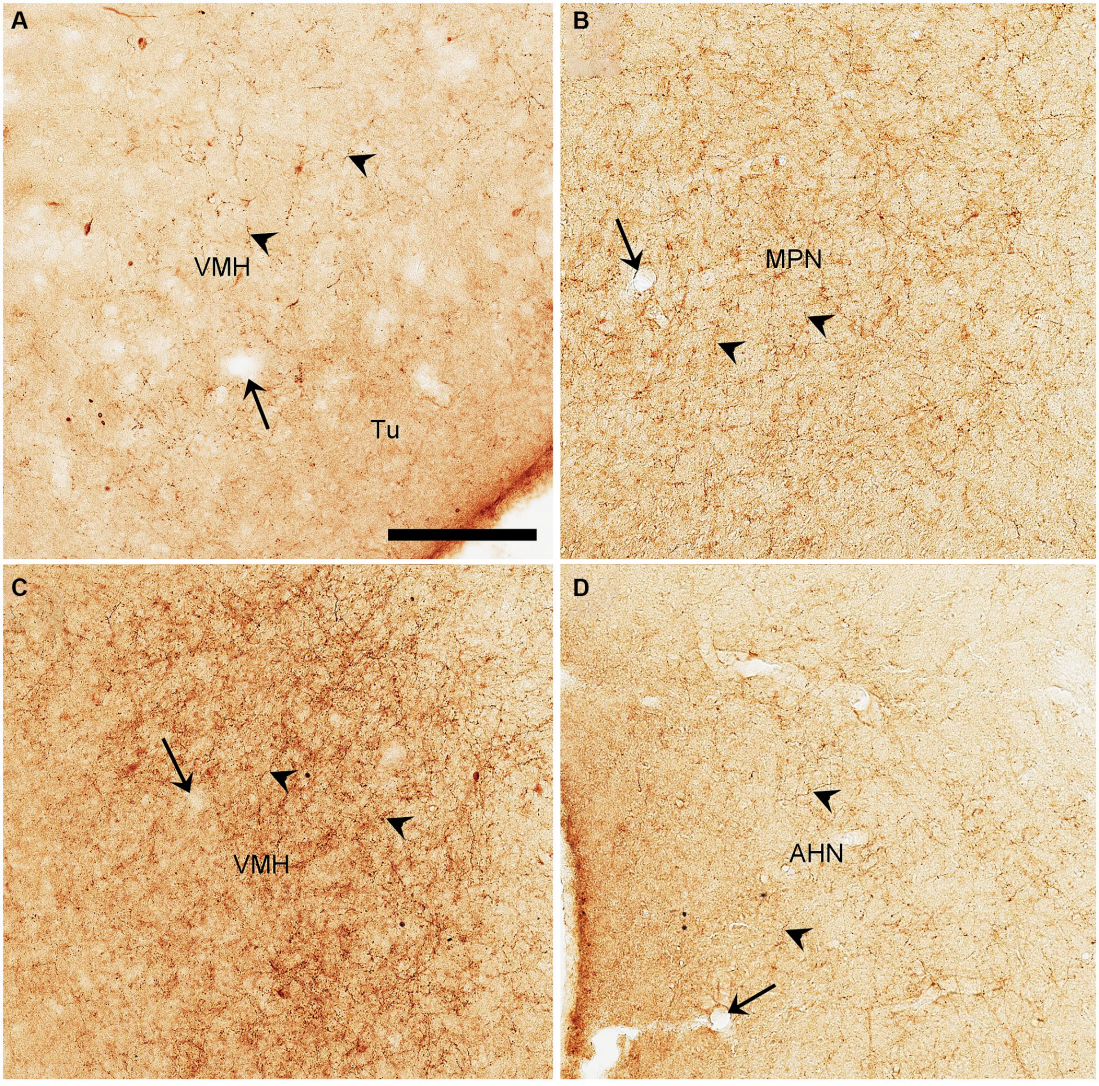
High magnification views of BDA labeled axon terminals in the rats. **(A)** The VMH from Figure 4I. **(B–D)** The MPN **(B)**, VMH **(C)**, and AHN **(D)** from Figures 5D–F, respectively. The arrows point to the same blood vessels as in the corresponding panels in Figures 4I, 5D,E,F. The arrowheads point to some dust-like BDA-labeled axon terminals. The density of the labeled axon terminals in panels **(A–D)** represents the semi-quantitative scores (+), (+++), (+++), and (++) in Table 1, respectively. Bar: 330 μm in panel **(A)** for all panels.

Semi-quantitative evaluation of the density of BDA-labeled axon terminals in main target regions of the PIL and PP reveals overall stronger projections of the PP vs. PIL (Table 1). Examples of stronger PP vs. PIL projections to the hypothalamic regions are shown in Figure 6. Sparse (Figure 6A) and dense (Figure 6C) terminal labeling in the VMH are obviously observed in the cases with PIL and PP injections, respectively. In addition, moderate terminal density is also seen in the MPN (Figure 6B) and AHN (Figure 6D) of the cases with PP injections. In contrast, only sparse or faint terminal labeling is detected in these hypothalamic regions in cases with PIL injections (see Figure 4).

**Table 1:** Semi-quantitative scores of BDA-labeled axon terminals in main target regions of the PIL and PP in rats.

	BDA injections in PIL	BDA injections in PP
ICx	−	+
ECT	+	+++
TeA	−	+
AStr	+++	+++
PuP	++	+++
GPe	+++	+++
CeM	++	+++
LaVm	++	+++
LaVl	+	+++
AAA	++	+++
BMA	++	+++
BMP	+	++
MeA	++	+++
MeP	++	+++
BST	−	+
AHN	−	++
VMH	+	+++
MPN	+	+++
RCh	+	++
SPf PSTN	+	++
ZI	++	++
LL PAG	−	+
SC	++	+++
IC	+++	++

^+^weak; ^++^moderate; and ^+++^strong or very strong labeling.

### 3.3 Borders and extent of the rat PP defined by its neurons projecting to VMH

**Figure 7:**
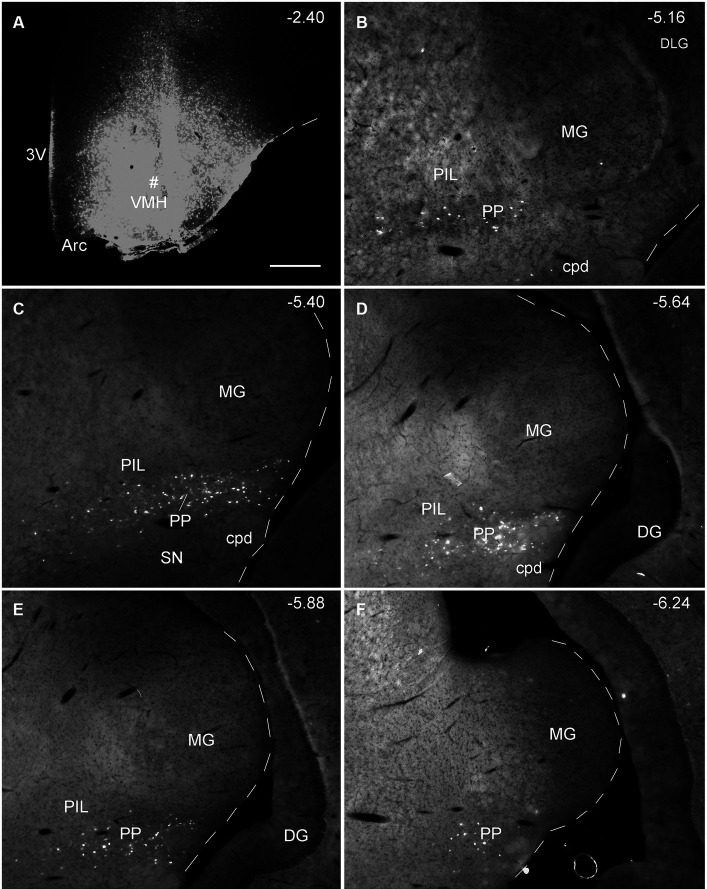
Extent of the rat PP revealed with retrograde tracer injection in the VMH. **(A)** One FG injection site (#) in the VMH. **(B–F)** Sequential coronal sections from the anterior **(B)** to posterior **(F)** levels showing the locations of FG-labeled cells in the PP following the injection. Note that no or only a few labeled cells are detected in the dorsally located PIL and nearby MG. The extent of the PP can be roughly identified and confirmed with the spatial distribution of FG labeled neurons. Approximate bregma coordinates are indicated at the top right corner of each panel. Bar: 500 μm **(A)** for all panels.

**Figure 8:**
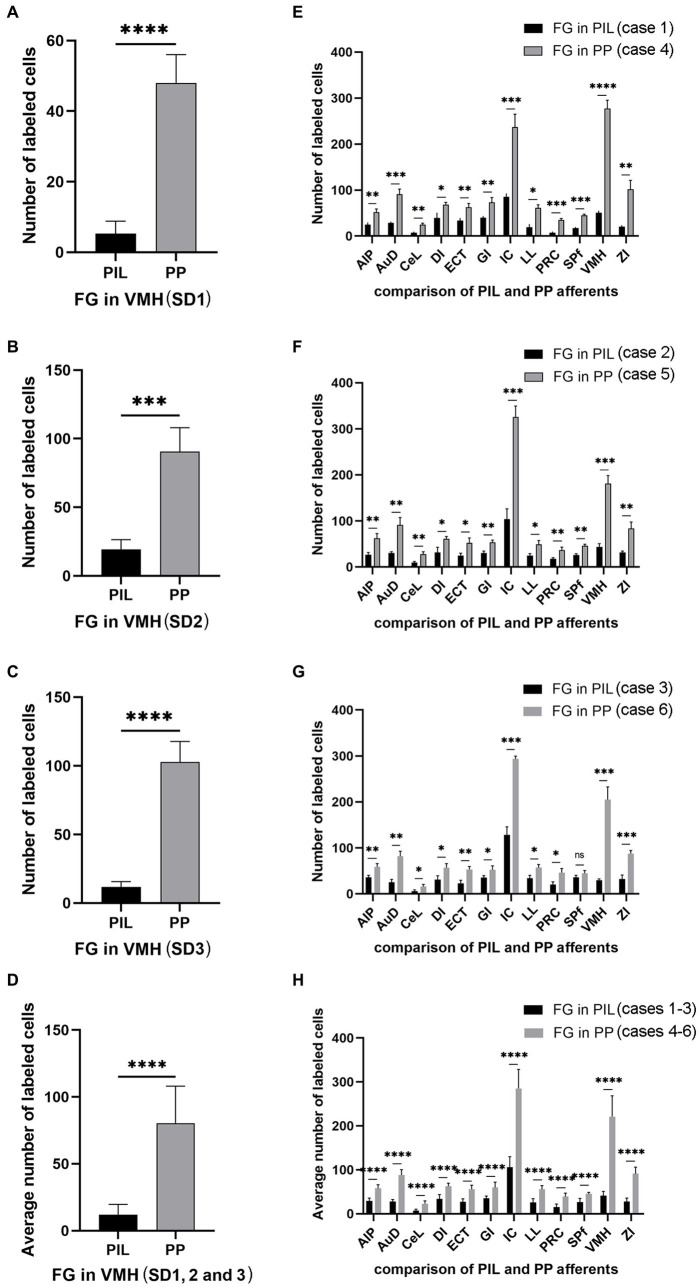
Quantification of FG labeled neurons in rat brains. **(A–C)** Number of labeled neurons in the PIL and PP resulted from FG injections in the VMH (cases SD1, SD2, and SD3). **(D)** Average number of the labeled neurons in the PIL and PP resulted from FG injections in the VMH (*n* = 3). **(E–G)** Number of labeled neurons in the main brain regions resulted from FG injections in the PIL (cases 1–3) and PP (cases 4–6). **(H)** Average number of the labeled neurons in the main brain regions resulted from FG injections in the PIL (*n* = 3) and PP (*n* = 3). ^*^p < 0.05; ^**^p < 0.01; ^***^p < 0.001; ^****^p < 0.0001.

Since the BDA anterograde tracing results indicate that the PP rather than the PIL strongly projects to the VMH, we wonder whether retrograde tracer injections in the VMH would lead to labeled neurons distributed only or mostly in the PP. This not only would further confirm BDA anterograde tracing results and but also get some insights into the borders and extent of the PP. As expected, following FG injections into the VMH (four cases), most of FG-labeled cells were observed in the PP with few in the PIL. As shown in Figure 7A, the FG injection centered in the VMH results in many FG-labeled cells in the PP along its anterior to posterior axis with few labeled cells in the overlying PIL and other adjoining regions such as the whole MG and SNL (Figures 7B–F). In other three cases, similar results were obtained (Figures 8A–C). Statistically, the average number of FG-labeled neurons in the PP is highly significantly greater than that in the PIL (*p* < 0.0001; Figure 8D). This result strongly supports the findings of anterograde and retrograde tracing studies described above.

### 3.4 Brain-wide afferent projections of the rat PIL and PP

**Figure 9:**
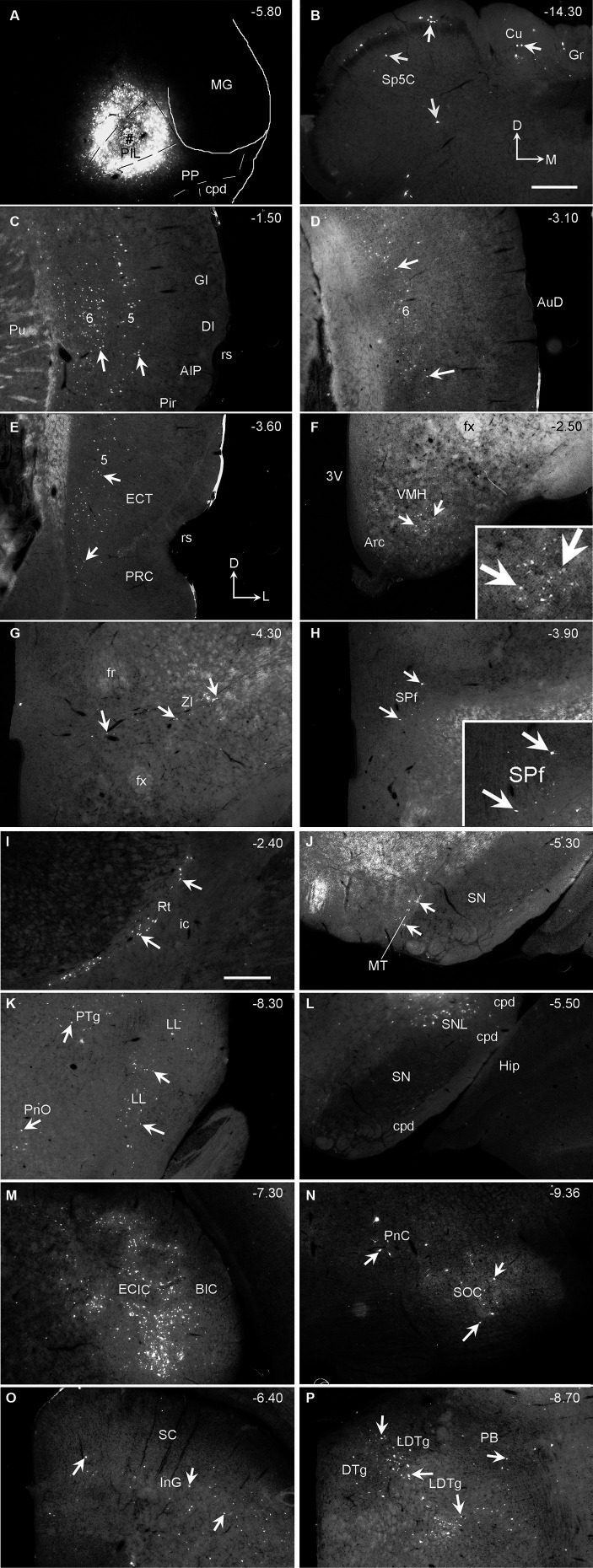
Afferent connections of the rat PIL revealed with FG. **(A)** One FG injection site (#) in the PIL without involvement in the PP. **(B)** Sparsely labeled neurons in the contralateral Gr, Cu, and Sp5C. **(C–P)** Distribution of ipsilaterally labeled neurons in layers 5–6 of the insular cortex (AIP, DI, and GI in **C**), layer 6 of the AuD **(D)**, layer 5 of the PRC and ECT **(E)**, and in the subcortical regions including the VMH **(F)**, ZI **(G)**, SPf **(H)**, Rt **(I)**, MT **(J)**, PTg, LL **(K)**, SNL **(L)**, IC **(M)**, SOC, PnC **(N)**, SC **(O)**, and LDTg **(P)**. Higher power views of the labeled neurons in the VMH **(F)** and SPf **(H)** are shown in the insets. The arrows in each panel point to some FG-labeled neurons. The orientation mark in panel **(B)** is for panel **(B)** only while that in panel **(E)** is for all other panels. Approximate bregma coordinates are indicated at the top right corner of each panel. Sequential sections through the injection site as well as corresponding low power views are shown in Supplementary Figure 1. Bars: 500 μm in panel **(B)** for **(A–H)**; 500 μm in panel **(I)** for panels **(I–P)**.

To reveal brain-wide afferent connections of the PIL and PP, we inject the retrograde tracer FG into the PIL (three cases) or PP (three cases) or both PIL and PP (four cases). FG injections concentrated in the PIL (e.g., Figure 9A; also see Supplementary Figure 1) result in some labeled neurons in the contralateral gracile nucleus (Gr), cuneate nucleus (Cu), and caudal part of the spinal trigeminal nucleus (Sp5C) of the medulla (Figure 9B) as well as in contralateral SC, IC, CnF, SPf, SOC and LL (data not shown). Other labeled cells are observed in the ipsilateral hemisphere. These include a limited number of cortical areas and many subcortical regions. For example, as shown in Figure 9, the FG injection (Figure 9A) produces labeled neurons in layers 5 and 6 of the insular cortex (Figure 9C), layer 6 of the dorsal association auditory cortex (AuD; Figure 9D), and layer 5 of the PRC-ECT (Figure 9E). The subcortical regions that contain FG-labeled cells mainly include the VMH (Figure 9F), ZI (Figure 9G), SPf (Figure 9H), reticular thalamic nucleus (Rt; Figure 9I), medial terminal nucleus (MT; Figure 9J), nucleus of lateral lemniscus (LL; Figure 9K), lateral part of substantia nigra (SNL; Figure 9L), IC (Figure 9M), superior olivary nuclei (SOC; Figure 9N), pontine reticular formation (PnC; Figure 9N), SC (Figure 9O), and LDTg (Figure 9P). Finally, some labeled cells are also scattered in the reticular formation of the pons and midbrain (data not shown). It is worth mentioning that no labeled cells were noted in the PBG.

**Figure 10:**
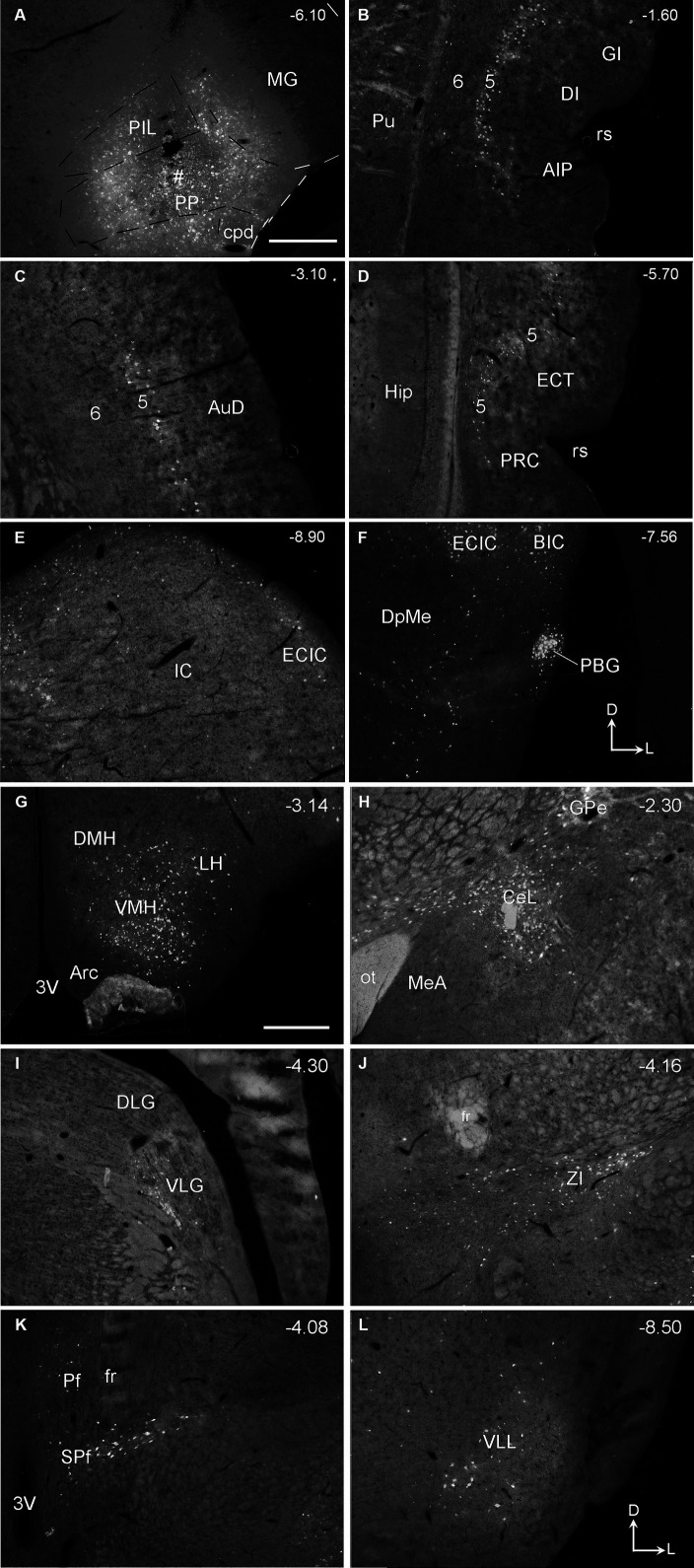
Afferent connections of the rat PP revealed with FG. **(A)** One FG injection site (#) in PP with some involvement in the PIL. **(B–L)** FG labeled cells in layer 5 of the insular cortex (AIP, DI, and GI in **B**), AuD **(C)**, PRC, and ECT **(D)**, and in the subcortical structures such as IC **(E)**, BIC and PBG **(F)**, VMH and LH **(G)**, GPe and CeL **(H)**, VLG **(I)**, ZI **(J)**, Pf and SPf **(K)**, and VLL **(L)**. Scattered cells were also observed in ipsilateral SNL, LDTg, SOC, PAG, and reticular formation of the medulla oblongata as well as in the contralateral Gr, Cu, and Sp5C (not shown). The orientation marks in panels **(F,L)** are for all panels. Approximate bregma coordinates are indicated at the top right corner of each panel. Bars: 500 μm in panel **(A)** for panels **(A–F)**; 500 μm in panel **(G)** for panels **(G–L)**.

Following FG injections mainly involved in the PP (e.g., Figure 10A), many labeled neurons are detected in layer 5 of the insular cortex (Figure 10B), AuD (Figure 10C), and PRC-ECT (Figure 10D) and the subcortical regions such as the IC (Figure 10E), PBG (Figure 10F), VMH (Figure 10G), and CeL (Figure 10H). A moderate number of FG-labeled cells are also seen in the medial part of ventral lateral geniculate nucleus (VLG; Figure 10I), ZI (Figure 10J), Pf and SPf (Figure 10K), and the ventral LL (VLL; Figure 10L). A few FG-labeled neurons are also found in the PB, SC, SOC, and PAG (data not shown) as well as in contralateral SC, IC, CnF, LL, SPf, SOC, Gr, Cu, and Sp5C. More FG-labeled neurons are observed in contralateral VMH and PBG (not shown). When FG injections are involved in both PIL and PP, labeled neurons are found in all the regions described above for both PIL and PP.

Quantification of the numbers of FG-labeled neurons in the main brain regions that originate the afferent projections to the PIL and PP (Figures 8E–H) confirms the findings described above. As shown in Figures 8E–G, the PP injections result in overall more FG-labeled cells in the regions originating the projections as compared to the PIL injections. These regions of origin include AIP, AuD, CeL, DI, ECT, GI, LL, PRC, SPf, ZI, VMH, and IC. Particularly, the latter two regions (VMH and IC) originate much stronger inputs to the PP as compared to the PIL (*p* < 0.001). Consistently, the average numbers of FG-labeled neurons in these brain regions are also significantly higher following PP injections as compared to PIL injections (*p* < 0.0001; see Figure 8H). These findings further confirm that there are significant differences in the intensity of afferent projections received by the PIL and PP.

### 3.5 Brain-wide efferent projections of the PIL and PP in mice

**Figure 11:**
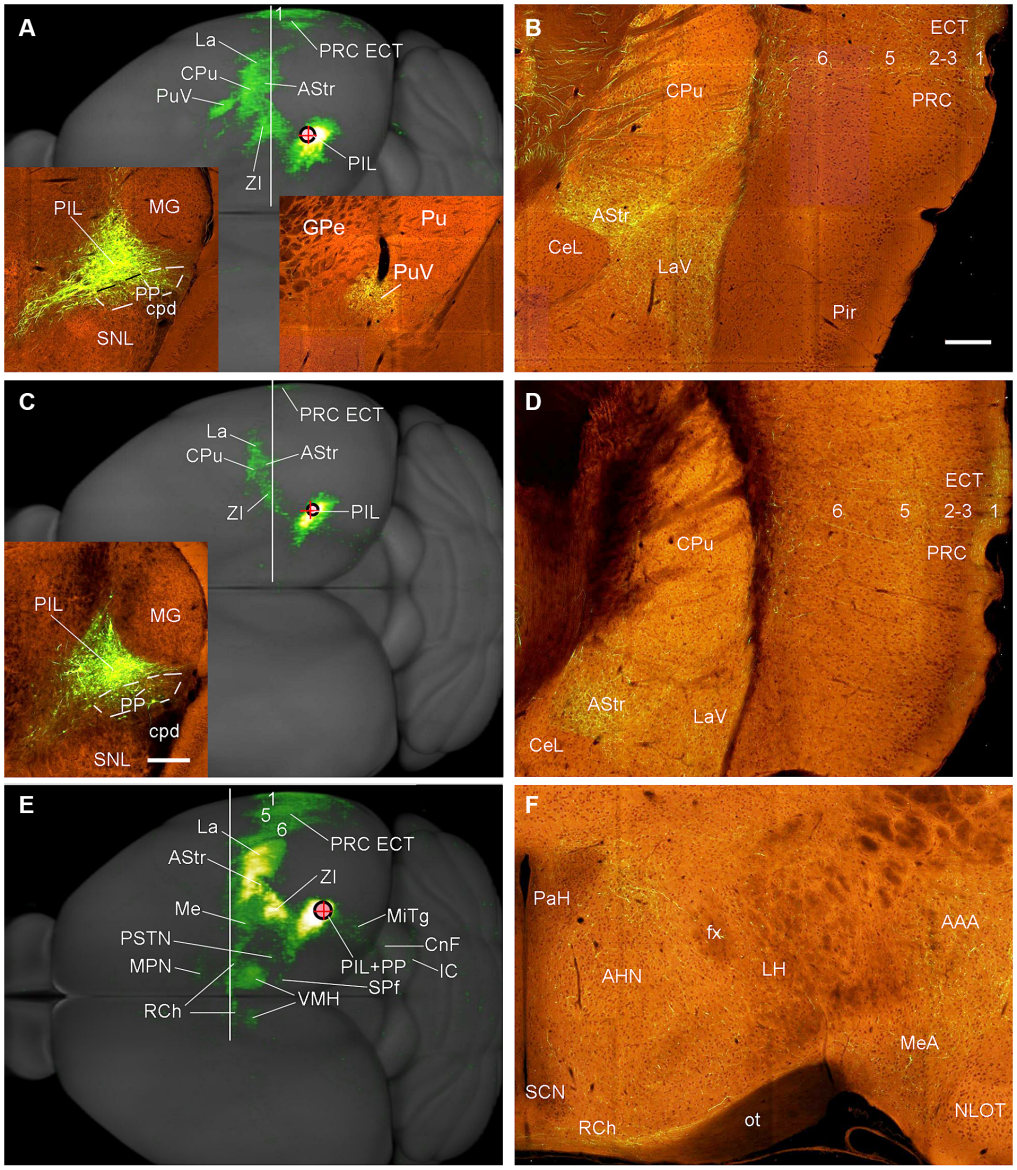
Efferent projections of the mouse PIL and PP revealed with Cre-dependent viral tracing in Cre-line mice. **(A,B)** Overall projection patterns of the PIL in one *Calb2*-Cre mouse. The tracer injection is restricted in the PIL without involvement in the underlying PP (See left inset in panel **A**) but involved in the LP-Pul and some large neurons in the SN (see Supplementary Figure 2). The main target regions of the PIL in this case include ECT (layers 1 and 5), La, AStr, CPu, ZI, as well as the ventral Pu (PuV in panel **A** and the right inset in panel **A**) with strong terminal labeling in the AStr and LaV **(B)**. One section through the level of the vertical white line is shown in panel **(B)**. **(C,D)** Overall projection pattern of the PIL in the second *Calb2*-Cre mouse. The tracer injection is restricted in the PIL without involvement in the PP (See the inset in panel **C**) and LP-Pul. The main target regions **(C)** of this PIL case are similar to the case shown in **(A)** except that no labeling is seen in the PuV, and overall labeling intensity is weaker. One section through the level of the white line is shown in panel **(D)**. **(E,F)** Overall projection patterns of the PIL + PP in one *Adcyap1*-Cre mouse. The tracer injection is involved in both PIL and PP (see the inset in panel **E**). The main target regions of this case include ECT (layers 1, 5, 6), La, AStr, CPu, ZI, as well as hypothalamic regions such as VMH (both sides; see panel **E**), AHN and RCh **(F)**. One section through the level of the white line is shown in panel **(F)**; other sections are shown in Figure 12. Bars: 200 μm in panel **(B)** for panels **(B,D,F)**; 280 μm in the inset in panel **(C)** for all insets.

To reveal the main efferent projections of the PIL and PP in mice, we have examined four cases with anterograde viral tracer injections restricted in the PIL and/or PP (from http://connectivity.brain-map.org). The viral tracers were delivered with iontophoresis. The overall efferent projection patterns of the PIL in two *Calb2*-Cre mice are similar to that revealed in the rats. Specifically, the main target regions of the PIL efferent projections include the ECT (mainly in layers 1 and 5), La, AStr, CPu, ZI, and CPu (Figures 11A–D) with strong terminal labeling in the AStr and LaV (Figures 11B,D). Weaker terminal labeling also exists in the MeA, MeP, BMA, PSTN, MiTg, and midbrain reticular formation (e.g., cuneiform nucleus, CnF). Almost no terminal labeling is seen in the hypothalamic regions such as the VMH, MPN, RCh, and AHN (Figures 11A,C). It is noted that the PIL injection shown in Figure 11A is involved in the LP (Pul) and SN (some large cells) (see Supplementary Figure 2), both of which are also *Calb2*-positive (see Figures 3A,B). In this case, the ventral putamen (PuV) also contains labeled axon terminals (see the right inset in Figure 11A).

**Figure 12:**
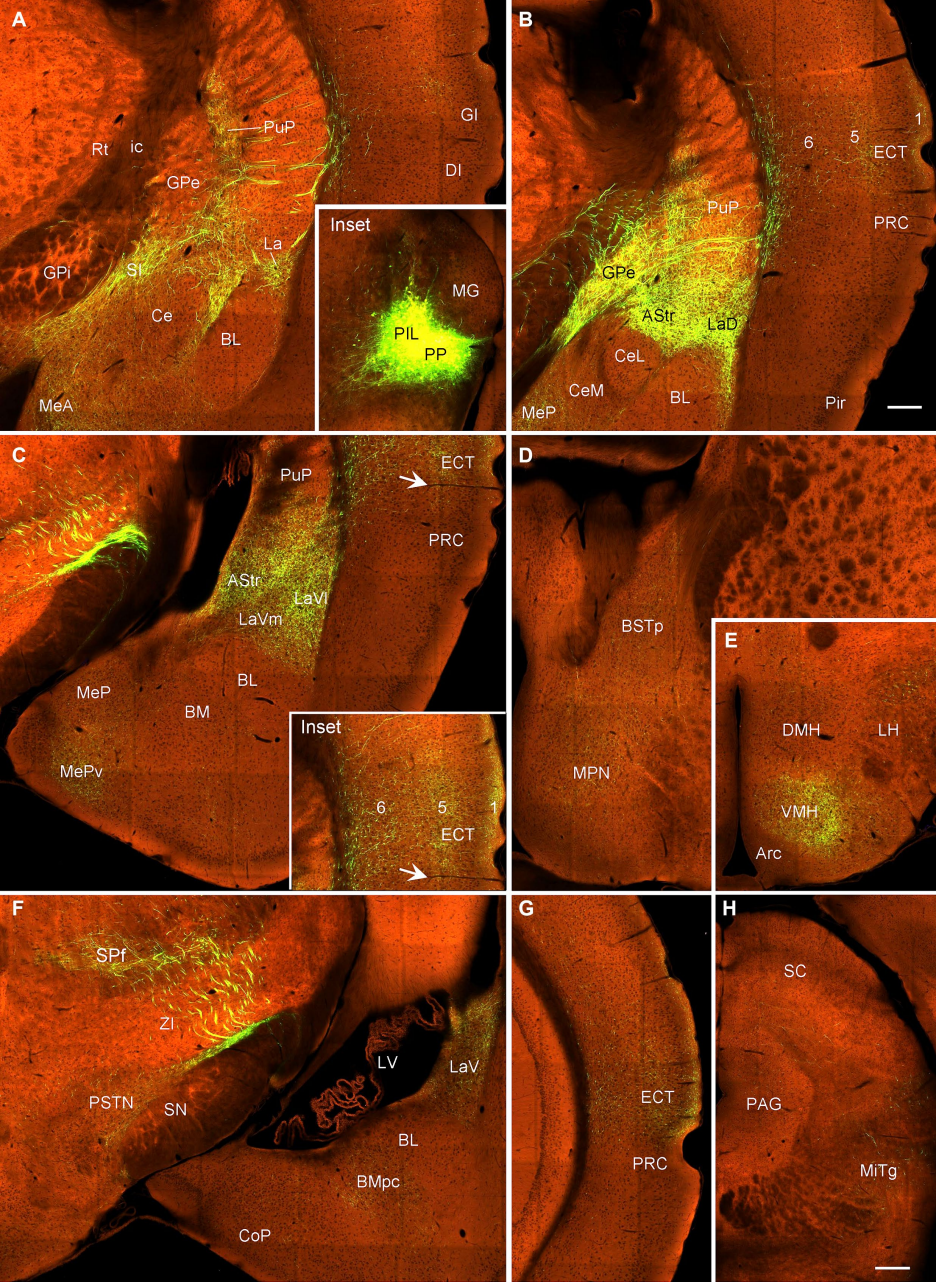
Efferent projections of the mouse PIL + PP revealed in *Adcyap1*-Cre mice. **(A–C)** Axon terminal distribution in the posteroventral striatum (PuP, GPe, and AStr), amygdala (La, MeA, and MeP) and in the insular (DI, GI in panel **A**) and anterior ECT (**B,C** and the inset in panel **C**) cortices after PIL + PP injection (Inset in panel **A**). **(D,E)** Terminal distribution in the BSTp, MPN, and VMH. **(F)** Terminal labeling in the SPf, PSTN, LaV, and BMpc. **(G)** Terminal labeling in the posterior ECT. **(H)** Weaker terminal labeling in the PAG and MiTg. Bars: 230 μm in panel **(B)** for all panels except panel **(H)**; 310 μm in panel **(H)**.

In contrast to the PIL injections, the injections involved in both PIL and PP (one *Adcyap1*-Cre and one wild-type mice) result in stronger terminal labeling in the ECT (in layers 1, 5, and 6), CPu, AStr, SPf, ZI, BST, AAA, LaV, BMA, BMpc, and Me (Figures 11E, 12A–H) compared to the PIL injections (Figures 11A–D). Strong terminal labeling is also seen in the VMH (Figures 11E, 12E) with weak to moderate labeling in other hypothalamic regions such as AHN, RCh, and MPN (Figures 11F, 12D). Labeled axon terminals are also found in the PSTN, PAG, SC, IC, BIC, LL, MiTg, and midbrain reticular formation (including CnF). Similar results are found in the wild-type case with the injection involved in PIL + PP except that weak terminal labeling is also seen in the LDTg (data not shown).

### 3.6 Confirmation of the projections from the VMH, LDTg, and Ce to PIL-PP in mice

**Figure 13:**
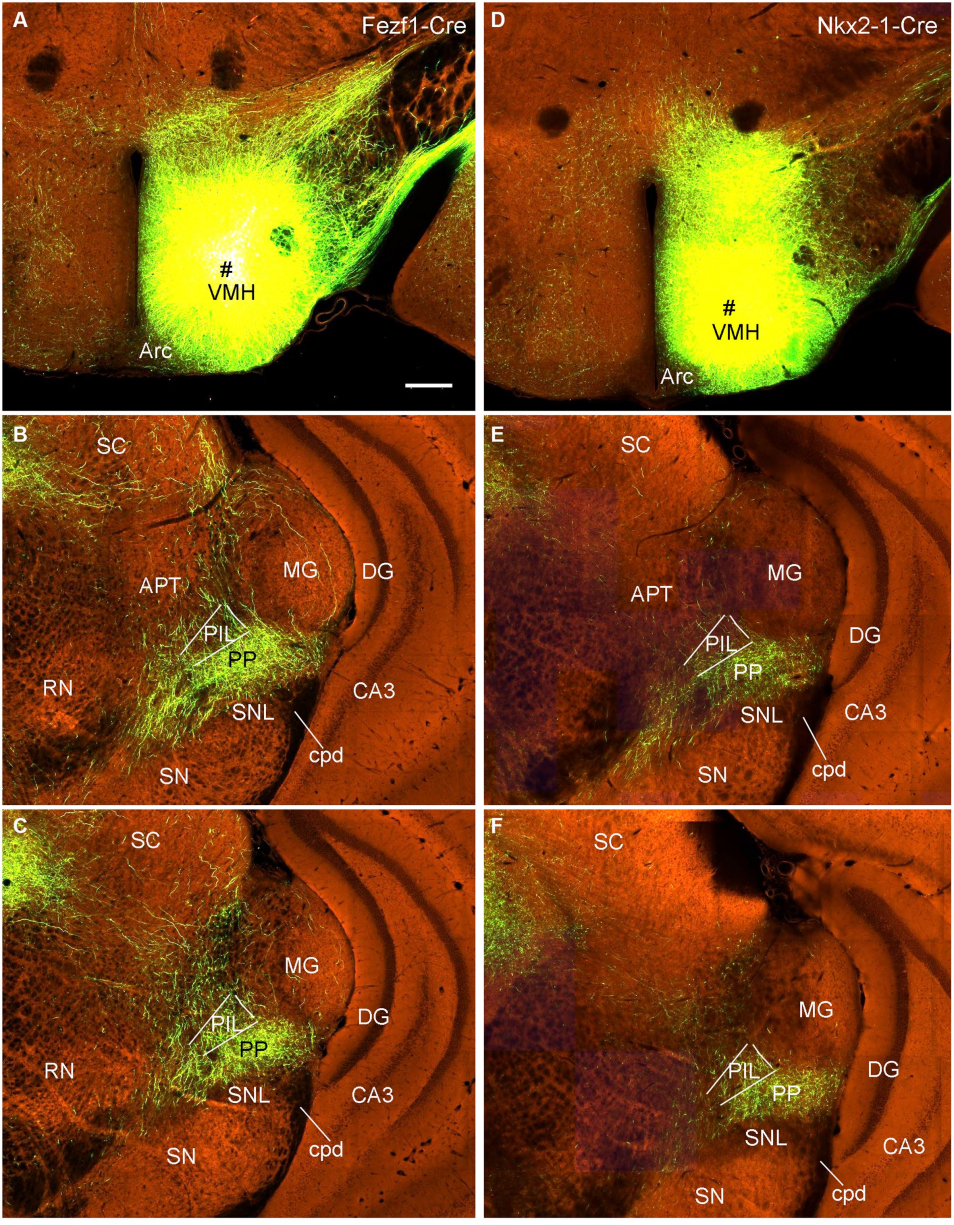
VMH projections to the mouse PP revealed with Cre-dependent viral tracing in Cre-line mice. **(A–C)** One viral tracer injection in the VMH of a *Fezf1*-Cre mouse **(A)** results in dense terminal labeling in the PP with weak labeling in the PIL **(B,C)**. **(D–F)** One viral tracer injection in the VMH of a *Nkx2-1*-Cre mouse **(D)** results in dense terminal labeling in the PP with weak labeling in the PIL **(E,F)**. Panels **(B,C,E,F)** represent anterior and posterior sections of each case. Bars: 280 μm in panel **(A)** for all panels.

To confirm the retrograde tracing results that the VMH, LDTg, and CeL contain labeled neurons following FG injections into the PIL and/or PP, we examined several cases with anterograde viral tracers restricted in these three structures (data derived from http://connectivity.brain-map.org). Like in the rats, the mouse VMH projects strongly to the PP as shown in Figure 13. Specifically, following anterograde viral tracer injections into the VMH of the Fezf1-Cre (two cases; e.g., Figure 13A) or Nkx2-1-Cre (two cases; e.g., Figure 13D) mice, many labeled axon terminals are found in the PP with only weak labeling in the PIL (Figures 13B,C,E,F). An anterograde tracer injection (see the inset in Supplementary Figure 3A) into the LDTg of the *Chat*-Cre mouse leads to labeled terminals mainly in the PIL with much fewer terminals in the PP (Supplementary Figures 3A,B). Another anterograde tracer injection (see the inset in Supplementary Figure 3C) into the Ce of a wild-type mouse produces labeled terminals mostly in the PP with much fewer terminals in the PIL (Supplementary Figures 3C,D).

## 4 Discussion

The PIL and PP appear to be evolutionally conserved structures since they not only exist in rodents, but also in monkeys and humans, and both structures strongly express *Calb1* (for calbindin-D28k) and *Calb2* (for calretinin) (e.g., Paxinos et al., 2012; Cai et al., 2019; Ding et al., 2022). The PIL-PP region was also reported to be enriched with tuberoinfundibular peptide of 39 residues (TIP39; Dobolyi et al., 2018). In this study, we have investigated brain-wide efferent and afferent projections of the PIL and PP following injections of the anterograde and retrograde tracers into the PIL or PP, respectively. The main connections of the PIL and PP have also been confirmed in wild-type and Cre-line mice (e.g., Figures 11–13; Supplementary Figure 3). In one hand, we have confirmed many connections of the PIL and/or PP reported in literature. In the other hand, we have uncovered some novel connections of the PIL and PP with potentially important implications (see below for discussion). By comparing the results with injections involved in the PIL and/or PP, we can distinguish some different connections between the PIL and PP. The most obvious differences between these two adjoining structures appear to be the connections with the VMH, IC, Ce, ECT, and PBG.

### 4.1 Leakage of neural tracers during injections

Leakage of neural tracers during stereotaxic injections is naturally expected in most neuroanatomical tracing studies, no matter which injection methods are used (including iontophoresis). For example, delivery of the viral tracers into the PIL via iontophoresis has produced tracer leakage into the LP-Pul (dorsal to the MG) and SNc in one case shown in Figure 11A of this study. The infected neurons in the SNc (see Supplementary Figure 2) have likely produced the terminal labeling in the PuV (see Figure 11A) since the PIL/PP (Figures 11C–F) and LP-Pul (see cases in http://connectivity.brain-map.org) do not send projections to the PuV. Therefore, more important things that are helpful for the interpretation of experimental results are the confirmation of the major results with other tracing methods and the comparison of the results between the cases with accurate and less accurate injection sites (with qualitative and/or quantitative analysis). For example, in this study, we have confirmed the anterograde tracing results of the PP-VMH connections with a retrograde tracing method (FG tracing) in rats (Figure 7) and with a Cre-dependent tracing method in mice (Figures 11, 12). We also compared the results from the cases with accurate injections in the PIL and with less accurate injections involved in both PP and PIL (Figures 11C–F). Additionally, we have quantified the labeled neurons and axon terminals in target regions and gotten consistent results (Figure 8; Table 1). Finally, we have also confirmed the retrograde tracing results of the projections from the VMH, LDTg, and Ce to the PIL and/or PP with anterograde viral tracing methods (Supplementary Figure 3; Figure 13). All these results support our major findings and conclusions.

### 4.2 Comparison with previous studies on the efferent projections of the PIL-PP

Some previous studies examined the efferent connections of the triangular region located ventromedial to the MG of the rats. In literature, this region was often treated as a single entity and either called PP (López and Carrer, 1982; Arnault and Roger, 1987) or PIL (Linke, 1999b), or paralaminar thalamic nuclei (Linke et al., 1999; Smith et al., 2006), or posterior intralaminar complex (Leithead et al., 2024), medial sector of the auditory thalamus (Wang et al., 2023). Based on cytoarchitecture, this complex region includes several smaller subregions including the PIL, PP, PoT, and MGm (Paxinos and Watson, 2013; Wang et al., 2020). It should also be pointed out that the PIL here is easily confused with the posterior intralaminar nucleus of thalamus, which includes centromedian and parafascicular nuclei (e.g., Ding et al., 2016). In some previous studies, when the anterograde tracers were placed in this PIL-PP region of the rats, labeled axonal terminals were observed in the La, Ce, Me, and BM of the amygdala and the PuPv and AStr of the striatum (LeDoux et al., 1990; Smith et al., 2019) as well as in the temporal and insular cortices (Linke et al., 1999; Smith et al., 2019) with no mention of the VMH. Retrograde tracing studies confirmed these main projections (LeDoux et al., 1990; Linke et al., 1999; Doron and Ledoux, 2000). In other previous studies, this complex region was reported to project to many hypothalamic regions including MPN, MPA, PaH, DMH, and LH as well as the LSN, PB, ZI, and PAG, in addition to the amygdaloid nuclei, PuPv, and temporal cortex mentioned above (Campeau and Watson, 2000; Keller et al., 2022). Some retrograde tracing studies confirmed these findings (Kita and Oomura, 1982; Chiba and Murata, 1985). In the present study, we have confirmed many of these efferent projections of the PIL-PP region. More importantly, we have revealed differential projections from the PIL and PP. Specifically, the PIL projects strongly to the CeM and faintly to the hypothalamic regions including the MPN, AHN, and VMH. In contrast, the PP sends much stronger projections to the ECT, VMH, MPN, AHN, and Me, and weaker projections to the CeM (see Figures 4–6). These results indicate that the PIL and PP have both common and differential efferent projections and thus may have differential functional correlation. The major and common output projections of the PIL and PP to the AStr, PuPv, and GPe may be related to their roles in motor modulation and deep brain stimulation for severe Parkinson’s disease (Stefani et al., 2007).

### 4.3 Comparison with previous studies on the afferent projections of the PIL-PP

In previous studies, projections from the SC and IC to the PIL-PP were examined with retrograde tracer injections in the PIL-PP region (Ledoux et al., 1987; Linke, 1999b; Mellott et al., 2014). Only a few studies systematically examined the afferent projections to the PIL-PP in rats (Arnault and Roger, 1987; Cservenák et al., 2017). These studies revealed labeled neurons in many cortical and subcortical regions. The cortical areas include ipsilateral insular, temporal and medial prefrontal cortices. The subcortical regions containing labeled cells are the ipsilateral SC, IC, SI, Ce, ZI, PB, VMH, and LPO, and the contralateral Gr, Cu, and Sp5C (Arnault and Roger, 1987; Cservenák et al., 2017). A recent study on mouse brains has obtained similar results (Cai et al., 2019). The present retrograde tracing study have confirmed the afferent connections of the PIL-PP from the temporal and insular cortices and the subcortical regions including ZI, SPf, VMH, Ce, SC, IC, PB, Gr, Cu, and Sp5C in the rats. Furthermore, we find additional afferent inputs from the SOC, LL, LDTg, and PuPv, which did not appear to be reported in rats (Arnault and Roger, 1987, Cservenák et al., 2017) but reported in mice (Cai et al., 2019). Finally, we find strong projections from the PBG (Figures 5H, 10F) and VGL-PP (Figure 10I) to the PP, and from the Rt (Figure 9P), MT (Figure 9J), and SNL (Figure 9L) to the PIL. To our knowledge, these projections were not reported in previous studies in rats and mice. Based on the distribution of FG labeled neurons, the PIL-PP region appears to converge a variety of multimodal information from auditory (AuD, IC, LL, and SOC), visual (SC, MT, VLG, and PBG), somatosensory (Gr, Cu, and Sp5C), motor (PuPv, GPe, and SNL), limbic (Ce and VMH), and multisensory (ECT and insular cortex) structures.

### 4.4 Differential connections and functional correlation of the PIL and PP

One of the main findings of the present study is the differential innervation of the hypothalamic regions from the PIL and PP. Specifically, the PP rather than the PIL provide significant inputs to many hypothalamic nuclei such as the RCh, MPN, and AHN, and strong projections to the VMH. Based on the ventral location of the PP, some previously reported “PIL” included the PP and were reported to project significantly to these hypothalamic regions (Cservenák et al., 2017; Dobolyi et al., 2018; Valtcheva et al., 2023). These hypothalamic regions are the effectors of a variety of social, stress, parental and maternal behaviors (López and Carrer, 1982, 1985; Hansen and Köhler, 1984; Factor et al., 1993; Dobolyi et al., 2018; Keller et al., 2022; Valtcheva et al., 2023; Leithead et al., 2024). Specifically, social interaction could elicit neuronal activity in the PIL-PP (Leithead et al., 2024). Auditory-PP-PaH pathway could mediate maternal oxytocin release induced by infant cries (Valtcheva et al., 2023). Sensory information from the rat pups could transmit to the MPO (MPN) and PaH of lactating mothers via the PP-MPO and/or PP-PaH pathways (Keller et al., 2022). The PP-MPO pathway is also involved in grooming in conspecific female rats during social interactions and thus plays a role in affinity social interactions (Keller et al., 2022). Consistently, c-fos expression in the PIL-PP neurons increases after lactation in maternal rats (Cservenák et al., 2010, 2013). Signaling through the “PIL”-PaH pathway contributes to the activation of oxytocin neurons in social contexts and the release of prolactin for milk expulsion; it also contributes to social recognition and belonging (Leithead et al., 2024). The glutamatergic “PIL” neurons respond to social and sexual behaviors in male and female mice and may modulate perceived social information to facilitate recognition and response to social stimuli (Leithead et al., 2024). Therefore, the PP-hypothalamic pathways likely play critical roles in all these behaviors. Additionally, the strong reciprocal connections between the PP and VMH (particularly the VMHvl) may be related to the functions of the PP in defensive arousal and sexual behavior (Hashikawa et al., 2017; Wang et al., 2023).

Many previous works focused mainly on the relay functions of the PIL-PP region, which links auditory inputs from the IC and auditory cortex to the output effector regions such as amygdala, suggesting this pathway as the substates for auditory fear conditioning (Romanski and LeDoux, 1992; Campeau and Davis, 1995; Campeau and Watson, 2000; Smith et al., 2006; Lanuza et al., 2008). However, the PIL-PP also receives stronger inputs from visual related structures such as SC, VLG, MT, and PBG, and thus could link visual information to the effectors such as the amygdala, hypothalamic, and striatum. For example, the PBG, which is found to project strongly to the PP in the present study, receives strong connections from the SC, VLG, prepositus hypoglossi complex, the locus coeruleus, the cuneiform nucleus, the periaqueductal gray, and the dorsomedial hypothalamic area (Baleydier and Magnin, 1979) and projects to the DLG and SC (Taylor et al., 1986; Turlejski et al., 1993; Deichler et al., 2020). Recently, Deichler et al. (2020) have suggested that the PBG may link visual information to defensive behaviors. The finding of strong PBG projections to the PP in the present study also suggests that the PBG may link visual information to social, maternal, and sexual behaviors.

Finally, there are also links between the PIL-PP and the cause of seizures (Benini and Avoli, 2006; Kita et al., 2016; Qi et al., 2022; Leithead et al., 2024). For example, causal role of calretinin-positive neurons in the PIL-PP was reported in temporal seizures (Bialer and White, 2010). The PIL-PP is an area containing dense calretinin-positive neurons that connect various brain regions implicated in epilepsy, such as the auditory and temporal cortices, ZI, and La (Linke, 1999a; Benini and Avoli, 2006; Kita et al., 2016). In addition, calretinin-positive neurons in PIL are activated during hippocampal seizures (Qi et al., 2022).

### 4.5 Summary

**Figure 14:**
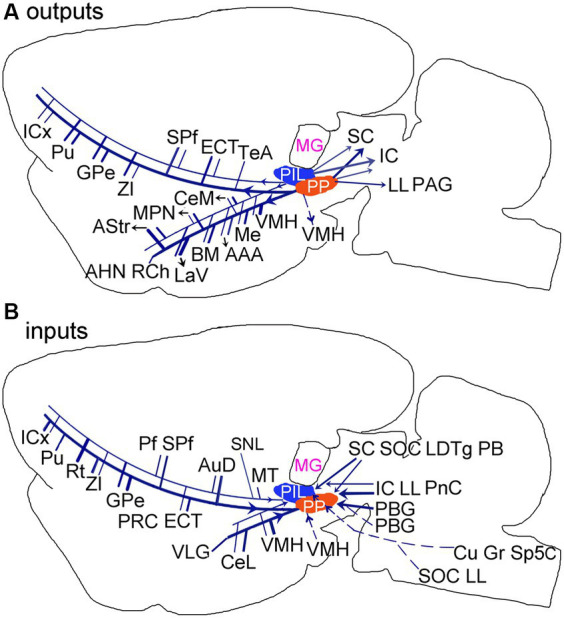
Summary of the main outputs **(A)** and inputs **(B)** of the rodent PIL and PP. Thick and thin lines indicate stronger and weaker ipsilateral projections, respectively. Dashed lines indicate contralateral projections. Overall, The PP has stronger connections with its connectional partners compared with the PIL.

The present study has systematically revealed brain-wide efferent and afferent projections of the PIL and PP and identified both common and different connections of these two adjoining structures (Figures 14A,B). The major common connections are those with the amygdala, striatum, thalamus and insular cortex. The main different connections are those with the hypothalamic regions, which the PP but not the PIL mainly projects to, and the PBG and VLG, which project to the PP but not to the PIL. The PIL-PP region is a hub for convergence of multimodal information and would associate different information to proper effectors such as amygdala (for auditory or visual fear conditioning), hypothalamic (for social, maternal, aggressive and sexual behaviors), and striatum (for motor modulation).

## Data availability statement

The original contributions presented in the study are included in the article/Supplementary material; further inquiries can be directed to the corresponding author.

## Ethics statement

The animal study was approved by the Institutional Animal Care and Use Committee of Guangzhou Medical University. The study was conducted in accordance with the local legislation and institutional requirements.

## Author contributions

H-RC: Formal analysis, Investigation, Writing – original draft. S-QC: Investigation, Supervision, Writing – review & editing. X-JX: Investigation, Writing – review & editing. X-QZ: Supervision, Writing – review & editing. R-ZM: Investigation, Writing – review & editing. GZ: Investigation, Writing – review & editing. S-LD: Conceptualization, Supervision, Validation, Writing – review & editing.

## Footnotes

^1^http://mouse.brain-map.org

^2^http://connectivity.brain-map.org

^3^http://www.brain-map.org

## Supplementary material

SUPPLEMENTARY FIGURE 1 An example of the extent of one FG injection in the PIL. **(A–C)** Sequential sections through the injection site to illustrate the location and anterior **(A)**–posterior **(C)** extent of the FG injection in the PIL. Corresponding low-power images are shown in the insets at the top right corners of each panel. The arrows point to the core of the injection site. The stars in the insets indicate the location of the MG. Bar: 500 μm in the panel **(A)** (for **A–C**).

SUPPLEMENTARY FIGURE 2 An example of neural tracer leakage during stereotaxic injections with iontophoresis method. **(A–C)** Three sequential sections through the PIL-PP of a *Calb2*-Cre mouse showing that the injection is mainly involved in the PIL with leakage into the LP and part of SNc. The SNc is indicated by the arrow in panel **(B)**. **(D)** A high magnification view of the PIL and adjoining SNc in panel **(B)**. The arrow points to the SNc, which is infected by the viral tracers. **(E)** A matched Nissl-stained section to panel **(B)** showing the location of the SNc, which contains larger neurons (arrow). Bars: 280 μm in the panel **(A)** (for **A–C**); 100 μm in panel **(D)**; 300 μm in panel **(E)**.

SUPPLEMENTARY FIGURE 3 Confirmation of retrograde tracing results with anterograde tracing methods. **(A,B)** One viral tracer injection into the LDTg (# in the inset in panel **A**) of a *Chat*-Cre mouse **(A)** results in sparse but clear terminal labeling in the PIL with weaker labeling in the PP **(A,B)**. **(C,D)** One viral tracer injection in the Ce of a wild-type mouse (# in the inset in panel **C**) produces dense terminal labeling in the PP with weak labeling in the PIL **(C,D)**. **(A,B)** and **(C,D)** represent anterior and posterior sections of each case. Bars: 140 μm in panel **(A)** for all panels; 280 μm in the inset in panel **(A)** for both insets.

## Glossary

3 V: Third ventricle
AAA: Anterior amygdaloid area
ac: Anterior commissure
Adcyap1: Adenylate cyclase activating polypeptide 1
AIP: Posterior agranular insular cortex
Arc: Arcuate nucleus of hypothalamus
AStr: Amygdalostriatal transition area
AuD: Dorsal auditory association cortex
bic: Brachium of inferior colliculus
BIC: Nucleus of brachium of inferior colliculus
BL: Basolateral amygdaloid nucleus
BMA: Anterior basomedial amygdaloid nucleus
BMP: Posterior basomedial amygdaloid nucleus
BST: Bed nucleus of stria terminalis
Calb1: Calbindin 1
Calb2: Calbindin 2
Ce: Central amygdaloid nucleus
CeC: Capsular subdivisions of the Ce
CeL: Lateral subdivisions of the Ce
CeM: Medial subdivisions of the Ce
CoA: Anterior cortical amygdaloid nucleus
CoP: Posterior cortical amygdaloid nucleus
cpd: Cerebral peduncle
Cu: Cuneate nucleus
DI: Dysgranualr insular cortex
Dk: Nucleus of Darkschewitsch
DLG: Dorsal lateral geniculate nucleus
DLTg: Dorsal lateral tegmental nucleus
DMH: Dorsomedial hypothalamic nucleus
DpMe: Deep mesencaphalic nucleus
DTg: Dorsal tegmental nucleus
ECIC: External cortex of inferior colliculus
ECT: Ectorhinal cortex
fr: Fasciculus retroflexus
GI: Granular insular cortex
GPe: External globus pallidus
GPi: Internal globus pallidus
Gr: Gracile nucleus
Hip: Hippocampus
ic: Internal capsule
IC: Inferior colliculus
InA: Intercalated nucleus of amygdala
InG: Intermediate gray layer of SC
InW: Intermediate white layer of SC
LaV: Ventral lateral amygdaloid nucleus
LaVl: Lateral parts of the LaV
LaVm: Medial parts of the LaV
LH: Lateral hypothalamic area
LPB: Lateral parabrachial nucleus
LPO: Lateral preoptic area
LP, LP-Pul: Lateroposterior-pulvinar nuclear complex
Me: Medial amygdaloid nucleus
MeA: Anterior subdivisions of Me
MeP: Posterior subdivisions of Me
MG: Medial geniculate nuclear complex
MGd: Dorsal subdivision of the MG
MGm: Medial subdivision of the MG
MGv: Ventral subdivision of the MG
MiTg: Microcellular tegmental nucleus
MPB: Medial parabrachial nucleus
MPN: Medial preoptic nucleus
MT: Medial terminal nucleus of accessory optic tract
LL: Nucleus of lateral lemniscus
NLOT: Nucleus of lateral olfactory tract
ot: Optic tract
ox: Optic chiasm
PAG: Periaqueductal gray
PaH: Paraventricular nucleus of hypothalamus
PB: Parabrachial nucleus
PBG: Parabigeminal nucleus
Pf: Parafascicular nucleus
PIL: Posterior intralaminar nucleus
Pir: Piriform cortex
PnC: Pontine reticular nucleus caudal part
PoT: Posterior thalamic nucleus
PP: Peripeduncular nucleus
PRC: Perirhinal cortex
PSTN: Parasubthalamic nucleus
Pu: Putamen
PuPv: Posteroventral putamen
RCh: Retrochiasmatic area
rs: Rhinal sulcus
Rt: Reticular thalamic nucleus
SC: Superior colliculus
scp: Superior cerebellar peduncle
SI: Substantia innominata
SN: Substantia nigra
SNc: Compact part of substantia nigra
SNL: Substantia nigra lateral part
SNr: Reticular part of substantia nigra
SOC: Superior olive nucleus
Sp5C: Spinal trigeminal nucleus caudal part
SPf: Subparafascicular nucleus
TeA: Temporal association cortex
Tppp3: Tubulin polymerization-promoting protein family member 3.
Tu: Tuberal nucleus
VLG: Ventral lateral geniculate nucleus
VLL: Ventral nucleus of lateral lemniscus
VMH: Ventromedial hypothalamic nucleus
VPMpc: Parvicellular part of ventroposterior medial nucleus
ZI: Zona incerta
