# Supplementary figures and images for: Comparison of the connectivity of the posterior intralaminar thalamic nucleus and peripeduncular nucleus in rats and mice
Source: Front Neural Circuits. 2024 Apr 26;18:1384621. doi: 10.3389/fncir.2024.1384621 (PMC11082296; doi:10.3389/fncir.2024.1384621)

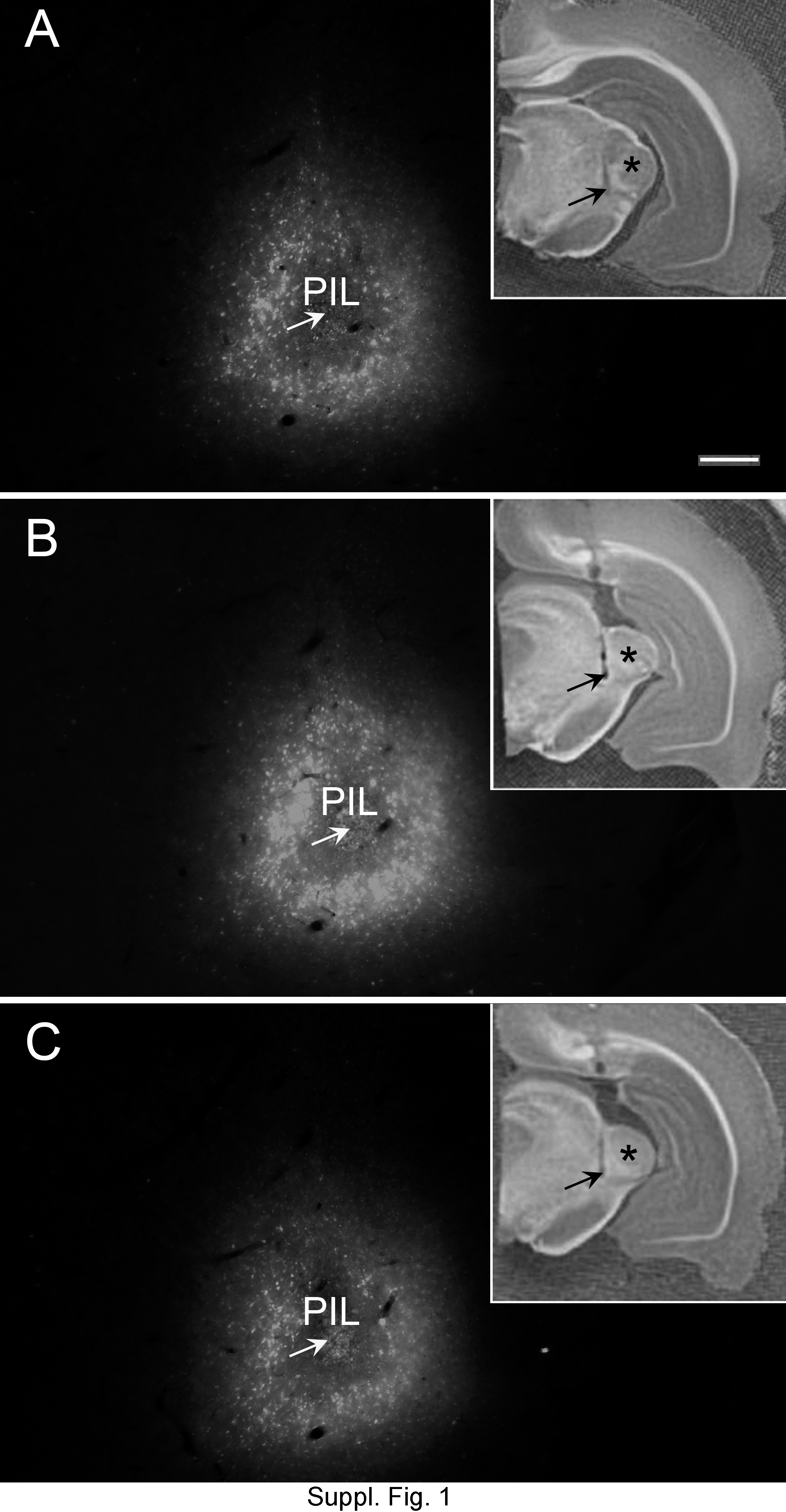

Supplement: SUPPLEMENTARY FIGURE 1 — An example of the extent of one FG injection in the PIL. (A–C) Sequential sections through the injection site to illustrate the location and anterior (A)–posterior (C) extent of the FG injection in the PIL. Corresponding low-power images are shown in the insets at the top right corners of each panel. The arrows point to the core of the injection site. The stars in the insets indicate the location of the MG. Bar: 500 μm in the panel (A) (for A–C). [file Image_1.tif]

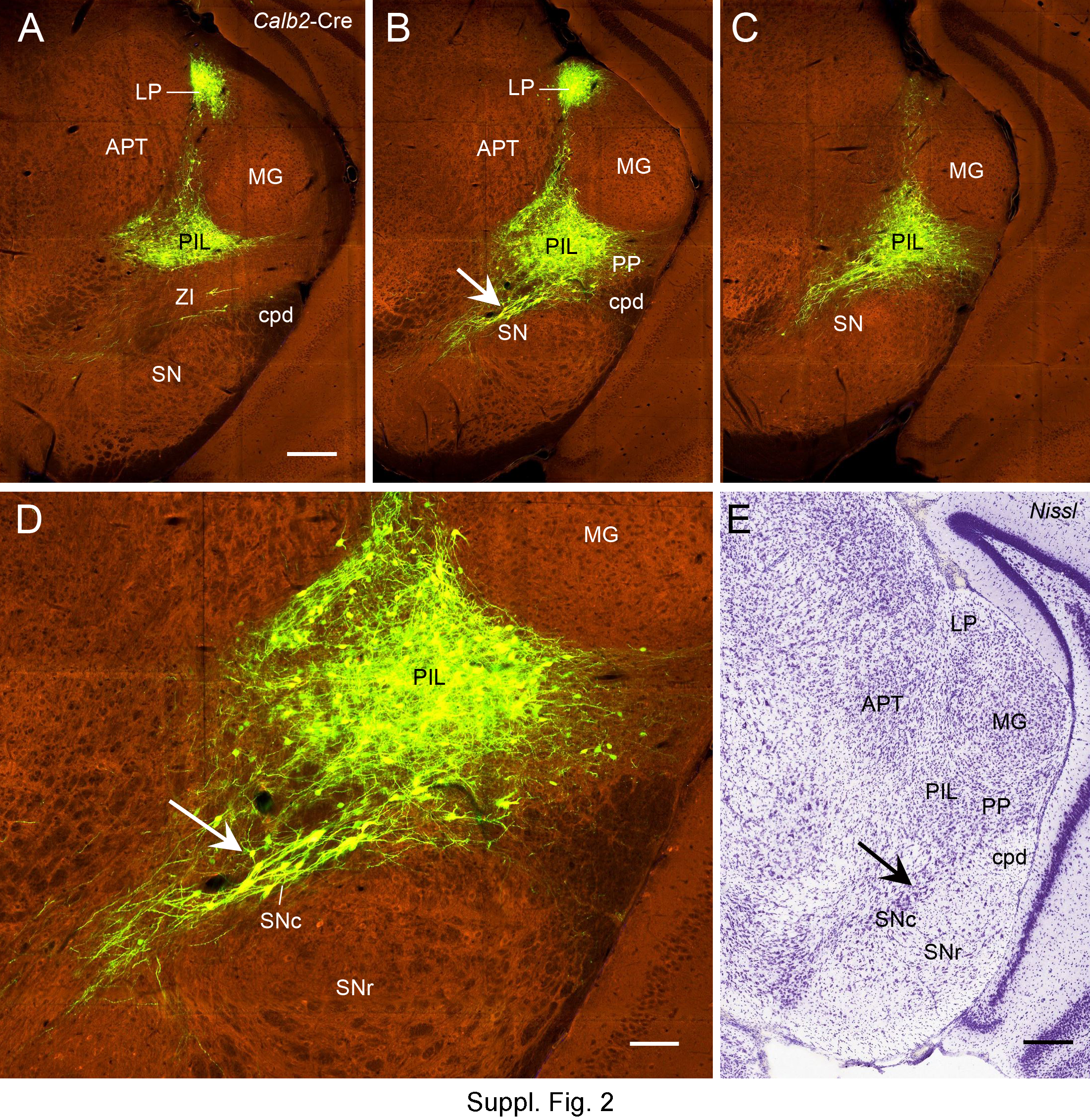

Supplement: SUPPLEMENTARY FIGURE 2 — An example of neural tracer leakage during stereotaxic injections with iontophoresis method. (A–C) Three sequential sections through the PIL-PP of a Calb2-Cre mouse showing that the injection is mainly involved in the PIL with leakage into the LP and part of SNc. The SNc is indicated by the arrow in panel (B). (D) A high magnification view of the PIL and adjoining SNc in panel (B). The arrow points to the SNc, which is infected by the viral tracers. (E) A matched Nissl-stained section to panel (B) showing the location of the SNc, which contains larger neurons (arrow). Bars: 280 μm in the panel (A) (for A–C); 100 μm in panel (D); 300 μm in panel (E). [file Image_2.tif]

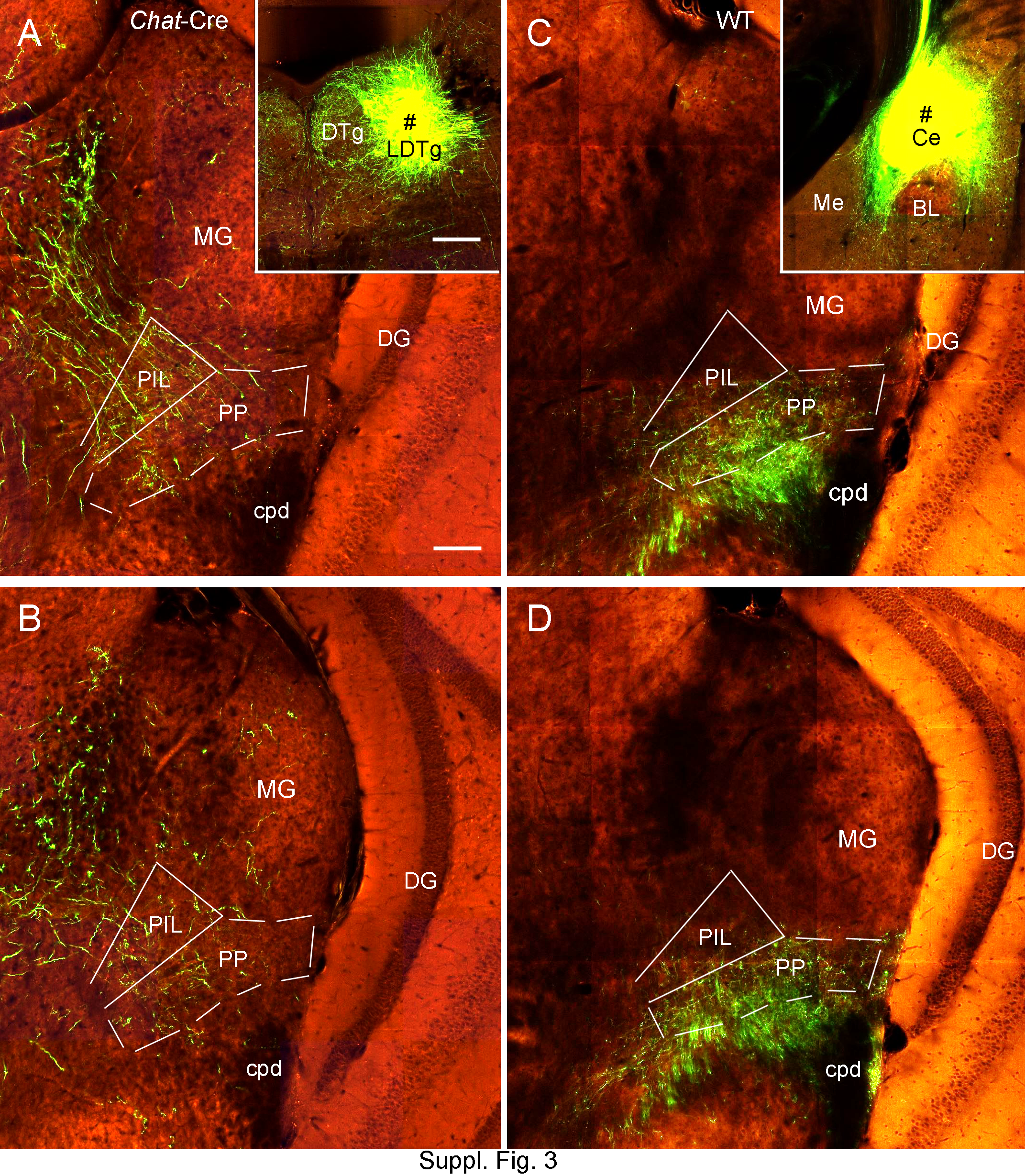

Supplement: SUPPLEMENTARY FIGURE 3 — Confirmation of retrograde tracing results with anterograde tracing methods. (A,B) One viral tracer injection into the LDTg (# in the inset in panel A) of a Chat-Cre mouse (A) results in sparse but clear terminal labeling in the PIL with weaker labeling in the PP (A,B). (C,D) One viral tracer injection in the Ce of a wild-type mouse (# in the inset in panel C) produces dense terminal labeling in the PP with weak labeling in the PIL (C,D). (A,B) and (C,D) represent anterior and posterior sections of each case. Bars: 140 μm in panel (A) for all panels; 280 μm in the inset in panel (A) for both insets. [file Image_3.tif]
